# Miniaturisation of Raman spectroscopy systems: from benchtop to backpocket

**DOI:** 10.1039/d5lc00836k

**Published:** 2026-03-18

**Authors:** Mike Hardy, Pooja P. Kanade, Emma Buchan, Pola Goldberg Oppenheimer, Cillian P. T. McPolin, Robert M. Bowman

**Affiliations:** a Smart Nano NI, Centre for Quantum Materials and Technologies, School of Mathematics and Physics, Queen's University Belfast Belfast BT7 1NN UK mhardy04@qub.ac.uk; b Advanced Nano-Materials Structures and Applications Laboratories, School of Chemical Engineering, University of Birmingham Birmingham B15 2TT UK; c Healthcare Technologies Institute, Institute of Translational Medicine Mindelsohn Way Birmingham B15 2TH UK; d Digital Catapult 8 Lanyon Place Belfast BT1 3LP UK; e Digital Catapult 101 Euston Road London NW1 2RA UK

## Abstract

The global portable spectroscopy market is rapidly expanding, with optical technologies composing the greatest market share. One prominent light-based technology is Raman spectroscopy, which confers benefits in terms of high selectivity and thus untargeted detection and multiplexing capabilities. Alongside this, plasmon-enhanced Raman technologies can provide ultra-low, even single molecule, detection. In this article, we analyse the current trends in the miniaturisation of Raman systems, including constituent components, evaluating current market needs, and providing a prospective on likely developments within Raman spectroscopy systems in the coming years. In particular, the progress from handheld Raman systems, which have surged in popularity since 2010 in a plethora of application spaces, to fully integrated on-chip Raman devices, is surveyed. Such palm-sized devices offer potentially easy integration into, for example, consumer white goods at home, and lightweight drone systems, and thus could transform the portable sensing landscape.

## Abstract 2

‘Progress of Raman systems from open-bench systems to miniaturised systems.’ Image generated with the assistance of Google Gemini.

## Graphical abstract

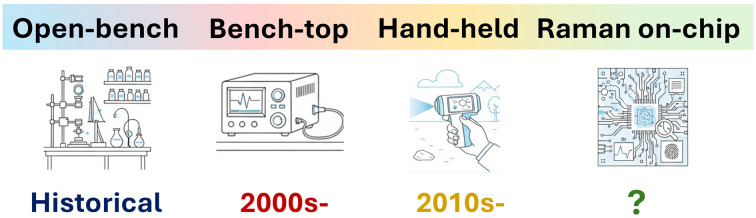

## 1. Introduction

‘Einstein was a giant’ with his head in the clouds but his feet on the ground, Richard Feynman reminds us. Those who built the Tower of Babel were less grounded and wanted to get their heads into the heavens. At 830 m (∼2720 ft), the Burj Khalifa (UAE) is currently the world's tallest building, by 2030 it will be overtaken by the Burj Jeddah (Saudi Arabia) and Oblisco Capitale (Egypt), which will both stretch 1000 m (∼3300 ft) skyward. Much of human endeavour has seen a fascination with an increase in size: ‘bigger is better’. However, not always so. Much like mobile phones in the 2000s, Raman spectroscopy systems started to shrink in size with an explosion of handheld Raman devices in the 2010s. While the trend for cell phones swiftly reversed – having the screen act as the tactile interface meant handsets started to get bigger again – Raman systems and related components have continued to downsize.

Increasingly, there is a need for portable sensing solutions as analytical analyses are needed away from university labs, clinics, to factory floors, home and outdoor environments. Such systems need to be easily manufactured, robust, with reasonable power consumption, as well as having easy operation. To this end, spectroscopic devices fit the bill, being potentially compact systems, at least carriable, and providing a wealth of information about a target sample. Optical techniques, in particular, have received increased attention, owing to their quick and easy implementation, and forming a part of the expanding global photonics-based technology market. Optical spectroscopy systems are particularly amenable to miniaturisation. Raman spectroscopy is one especially prominent optical spectroscopic technique making inroads into commercial adoption, which has with it advantages over companion technologies.

Raman scattering is the inelastic interaction of light *via* molecular vibrations and carries with it well-known advantages in terms of high analytical specificity (selectivity), made possible by inherently sharp peaks, and in the modern era, facile sample preparation. Like infrared (IR) absorption spectroscopy, Raman spectroscopy is typically non-destructive, but with the added advantage of being insensitive to water, which is a very weak Raman scatterer, thus enabling a wide array of applications. Most sensors take indirect measurements^1^*i.e.* on an ion or molecular species other than the target (from which the target analyte can subsequently be identified and/or quantified) but Raman spectroscopy can be performed directly or indirectly meaning with or without an intermediary functionalisation molecule at the sensor surface.^2^ Pertaining to the spectroscopy aspect of the technique, further advances in lasers, optics, filters, spectrometers and detectors have made Raman, while once expensive and difficult to perform well, now accessible. Moreover, the marriage with plasmonic enhancing media in the context of surface enhanced Raman spectroscopy (SERS) has made trace level detection possible, albeit debate remains over SERS' place as a properly quantitative analytical technique. Nevertheless, Raman and SERS have made tremendous progress in recent decades. However, much of this advancement has been in the laboratory where Raman spectroscopy and its various derivative techniques have been applied in large research spaces.

The market for handheld Raman systems has boomed in recent years (say, 2010s – onwards) driven by applications outside of the laboratory, where spectra can be rapidly converted into actionable information.^3^ Alongside this, small devices, including microfluidic devices, and small sensing chips as in SERS sensors, have been in development. These advancements not only serve the portability requirement but offer a smaller form factor, and thus also constitute the additional advantages of lower material cost and a smaller bill of materials (BOM).^4^ It is a good question, however, just how integrated, how miniaturised, Raman systems will go, what is possible with modern technology and what industry needs.

### 1.1. Article outline

**Fig. 1:**
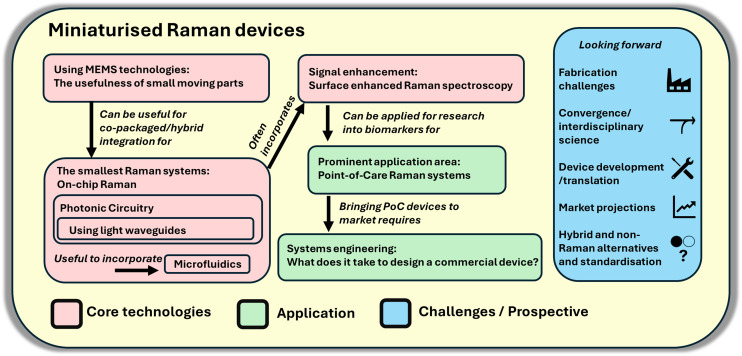
Diagrammatic summary of topics covered in this review.

**Fig. 2:**
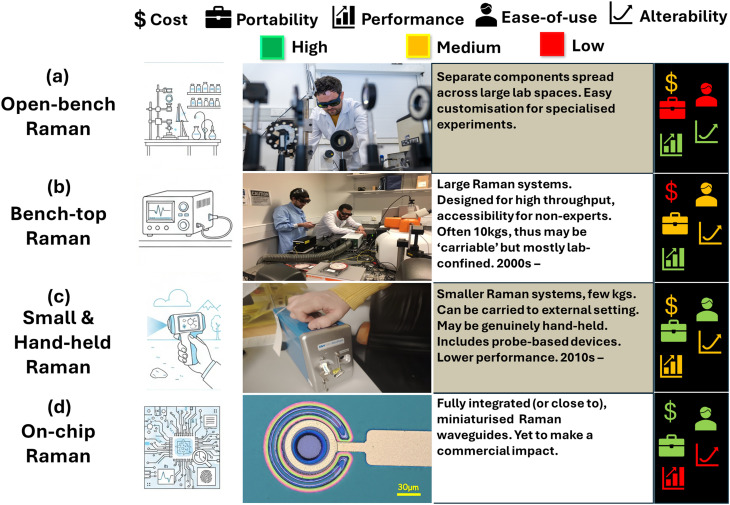
Different kinds/sizes of Raman systems: (a) large open-bench systems confined to labs, (b) large but portable Raman systems, often permanently located in labs, with easy operation, (c) hand-held portable Raman systems of a few kgs, typically, which can give rapid, actionable results, (d) fully miniaturised Raman systems, often highly integrated spectrometers, optics, electronics. Possibly palm-sized. Key (far-right) indicated relative cost, portability, performance, ease-of-use, and ability to modify the system (‘alterability’), where green = high, yellow = moderate, red = low. Image partially generated with the assistance of Google Gemini. Photo credits: Fig. 2 (a) (second column): Conor McKernan, Comms Team, Queen's University Belfast; (b) Dr Liam Kelleher, School of Chemical Engineering, University of Birmingham. (d) Dr Katie F. Cavanagh, Yelo Ltd, and Smart Nano NI, Queen's University Belfast. Image in (d) is a vertical-cavity surface-emitting laser (VCSEL) from the Smart Nano NI labs. Images in first column generated with the assistance of Google Gemini.

This article covers a range of connected areas within Raman device miniaturisation from small moving components, heading towards full on-chip integration, enhancement for trace analyte analysis, and applications (Fig. 1). In the opening sections, this article outlines some of the background in Raman system development from the discovery of the Raman effect to current laboratory-confined systems (section 2) and more recent hand-held devices (section 3). There is a focus on probe-based Raman devices, a notable system accessory, and efforts in miniaturisation of spectrometers. Subsequently, the impact of micro-electro-mechanical systems (MEMS), an emerging area involving the fabrication of tiny moving, electrical parts, in Raman is outlined for various components in small Raman systems (section 4). The discussion then turns to the smallest Raman devices, on-chip Raman and Raman waveguides (section 5), often fully integrated systems that allow Raman spectroscopy to be done on reduced scales, and may incorporate nanostructures facilitating surface enhanced Raman spectroscopy (SERS), which is outlined in section 6. We then survey the most prominent application area for small Raman systems: point-of-care (PoC) Raman devices, which are portable systems most readily associated with healthcare applications, often remote from the clinic, including an overview of microfluidics (section 7). Our analysis concludes with a look at points pertaining to Raman systems engineering (section 8) – how do we actually make a marketable Raman device? –, and a forward-looking assessment of the current portable Raman market and challenges on the horizon in future developments in section 9. The review therefore aims to summarise the key developments in the push for smaller and smaller systems to date (Fig. 2) and offer some prospective on the way forward in the coming years.

## 2. History of Raman systems: early developments to benchtop systems

The first Raman system had primitive elements. C.V. Raman used coloured glass filters and a readily available light source – the Sun! Similarly, colour changes were initially detected by eye. He subsequently used mercury lamps and a spectrograph, recording the Raman lines on a photographic plate. Later, uviol glass was used for UV frequencies (<250 nm). As a technique, Raman spectroscopy was viewed as the poorer sibling of infrared (IR) absorption spectroscopy, a similar technique in many ways but involving a change in electric dipole moment (rather than the electrons' polarisability as in Raman scattering). Until the middle of the 20th century, Raman analyses on aqueous samples needed multiple distillations for the inherently low Raman signal not to be overwhelmed by fluorescence signal from interferents. Thus, IR spectroscopy was the technique of choice, despite long measurement times.^5^ This kind of statement might appear strange today because Raman is often highlighted as ideal for liquid analysis where water has a low Raman signal. World War Two brought with it developments in IR detectors, which benefited IR spectroscopy for analytical analyses.^6^ Subsequently, the availability of high-powered, monochromatic light revived Raman spectroscopy as an analytical technique, tallied with improving detection elements.^6,7^

### 2.1. Detectors, filters, software

**Fig. 3:**
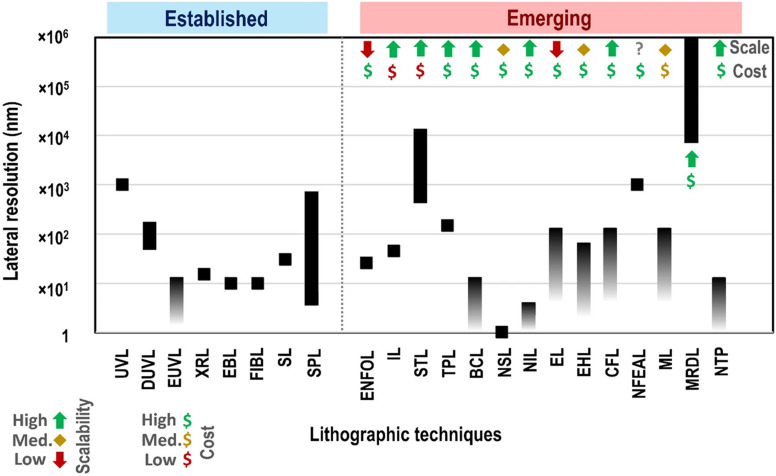
Emerging lithographic techniques. UVL = conventional UV lithography, DUVL = deep ultraviolet lithography, EUVL = extreme ultraviolet lithography, XRL = X-ray lithography, EBL = electron beam lithography, FIBL = focused ion beam lithography, SL = soft lithography, SPL = scanning probe lithography, ENFOL = evanescent near-field optical lithography, IL = immersion lithography, STL = stereolithography, TPL = two-photon lithography, BCL = block copolymer lithography, NSL = nanosphere lithography, NIL = nanoimprint lithography, EL = edge lithography, EHL = electrohydrodynamic lithography, CFL = capillary force lithography, NFEAL = near-field electrospinning-assisted lithography, ML = magnetolithography, MRDL = magnetorheological drawing lithography, NTP = nanotransfer printing. Full details of all techniques in Stokes *et al.* Adapted/reproduced from ref. 8 with permission from Springer Publishing under Creative Commons CC-BY-4.0, K. Stokes *et al. Discover Nano*, **18**, 153, copyright 2023.

Of course, these early happenings in Raman were on a large, lab-bench scale. Lasers were metre-sized gas-based tubes and spectrometers were similarly sized bulky instruments with large diffraction elements and long optical paths. Alongside this, improvements in detection elements, meaning small and more densely packed pixels for higher resolution and smaller parts, improvements in sensitivity (quantum efficiency) *via* better access to the photoactive area *e.g.* backside illumination, and large dynamic ranges available with less noise. Similarly, faster read-out speeds have enabled fast Raman imaging techniques, or high throughput Raman applications otherwise. Charged-coupled device (CCD) detectors have been used as the detector element of choice for Raman systems, owing to their greater sensitivity over their complementary metal oxide semiconductor (CMOS) counterparts, having replaced photographic plates and photomultiplier tubes in the 80s. CCDs have improved in many ways since their appearance, where lower noise (deep depletion technology) and the integration of small thermoelectric cooling systems have increased sensitivity. Similarly, developments in lithographic processes have permitted smaller fabricable elements^8^ (Fig. 3). CMOS performance, however, has been catching up (SI section: Table S1), and has been critical as far as reducing the size of Raman systems, where CMOS detectors are cheaper, less power-hungry and can be easily integrated on chip. Further, CMOS is mechanically simpler, thus more shock resistant *i.e.* high ruggedness for portable detectors, and impervious to CCD ‘blooming’ (overspill of electrons into adjacent pixels). Further still, the row-by-row readout in CMOS may be useful for some applications, such as hyperspectral Raman imaging. Hyperspectral imaging, which combines spatial and/or temporal information along with the spectral data to form multidimensional data ‘hyper-cubes’, has received intense research effort over the past 10 years, although more often in IR spectroscopy.^9^

More recently, developments in Raman technology have included better optical filters, improving sensitivity with higher bandpass transmission percentages and sharper cut-offs. The latter has been especially useful in low-frequency (THz) Raman applications, which is useful for garnering structural information from various samples, for instance 2D material analysis.^10^

Benchtop Raman instruments started to appear in the 90s and into the 2000s as systems, while still lab-based and on the 10 kgs-scale, were now becoming more integrated. Such systems adopted more user-friendly software with graphical user interfaces (GUIs) that ensured Raman systems were accessible to a wide-range of scientists, not only spectroscopy specialists. Moreover, associated software packages for data post-processing, which can perform machine learning tasks and have sizable spectral libraries, are evermore available. Inscore (2011), for example, built up a significant Raman spectral library of 150 illicit drugs.^11^ This kind of data bank, for instance can be useful for law enforcement, *via* use of a simple spectral matching algorithm as part of software on a portable Raman sensor at a crime scene. Similar libraries are needed for enhanced Raman analyses, where spectral peaks may shift position slightly, new peaks appear^12^ or those measured in complex matrices with confounding substances,^13^ for example, food debris in an analysis of oral fluid without filtration or centrifugation.^14^

Benchtop systems, while ‘portable’, are intended primarily for laboratory use and remain popular for high-end Raman analyses and routine, high-throughput analytical analyses. True hand-held Raman instruments, of the order of kgs, appeared subsequently, gaining traction from 2010s onwards, and have continued to be popular. One of the interesting developments are the number of accessories now available in benchtop and handheld Raman systems. This includes *xyz* translation stages with colinear cameras for Raman microscopy and various adapters for different kinds of samples, *e.g.* cuvettes and SERS media.

### 2.2. Raman probe systems

**Fig. 4:**
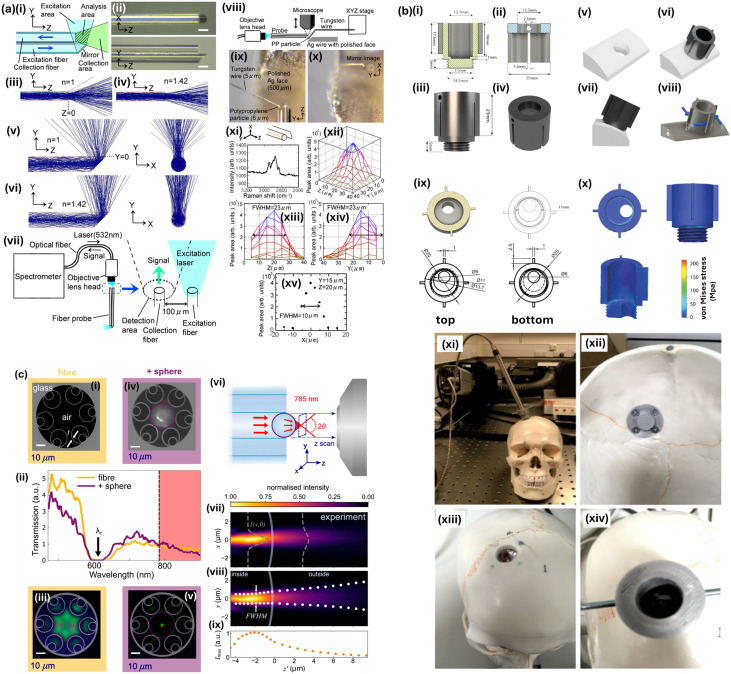
Raman probe systems. (a) Ultrafine fibre Raman probe with high spatial resolution and fluorescence noise reduction. (i) A schematic illustration of the probe. (ii) Photographs of the probe. Scale bars indicate 30 μm. (iii) and (iv) Simulated trajectories of light emitted from the collection fibre into air (*n* = 1) and ethylene carbonate (*n* = 1.42), respectively. (v) and (vi) Simulated trajectories of light emitted from the excitation fibre into air and ethylene carbonate, respectively. (vii) Experimental setup for Raman spectroscopy using the probe. (viii–xv) Measurements of spatial resolution. (viii) Experimental setup for determination of spatial resolution of the probe. (ix) Microscopic observation to confirm the position of a polypropylene (PP) particle relative to the probe in the *YZ* plane. (x) Microscopic observation to confirm the position of the PP particle relative to the probe in the *XY* plane. (xi) Raman spectrum of the PP particle measured with the probe. The spectrum was averaged over two spectra taken with incident power of 20 mW and 60 s. (xii) Area of the peak of PP plotted against the *YZ* plane. (xiii) Peak area profile plotted against the *Z* axis made by projection of the profile shown in (xii). (xiv) Peak area profile plotted against the *Y* axis made by projection of the profile shown in (xii). (xv) Peak area profile plotted against the *X* axis at (*Y*, *Z*) = (15, 20). Adapted/reproduced from ref. 20 with permission from American Chemical Society, Yamanaka *et al. J. Phys. Chem. C*, **120**, 2585–2591, copyright 2016. (b) Intracranial probe design and skull model for Raman spectroscopy in traumatic brain injuries. (i) Clip is used to aid device positioning and probe alignment: (i) cross-sectional analysis of the Raman probe housing and thread. (ii) Cross-sectional analysis of the clip and baffle slots. (iii) Tightening and alignment clip fitted to the probe housing. (iv) Aerial view of the clip. (v–viii) *In situ* analysis of the bolt: (v) approximation of the frontal bone at Kocher's point. (vi) and (vii) Top and side views of simulation. (viii) Implementation of load and constraints. (ix) Section analysis of the housing (left) to demonstrate the internal ledge and drawing of top view of the probe housing (right), with the dimensions of the ledge indicated. (x) Points of high stress in ABS plastic (acrylonitrile, butadiene, styrene) and fracture propagation at the bolt joint. The legend is indicative of the von Mises stress developed in the structure. (xi–xiv) Development of a skull model. (xi) Fully assembled model. (xii) Sample holder internal to the skull. (xiii) Sample holder at Kocher's point. (xiv) Medical device *in situ*. Adapted/reproduced from ref. 18 with permission from American Chemical Society under Creative Commons CC-BY-4.0, Mowbray *et al. ACS Biomater. Sci. Eng.*, **7**, 1252–1262, copyright 2021. (c) Imaging and transmission of a nested antiresonant fibre (NANF) and fibre-probe. (i) SEM images of an unmodified NANF facet. (ii) Transmission of NANF with and without microlens. (iii) Image of NANF facet, with a super continuum laser coupled in. (iv) SEM image of the fibre–microlens probe. (v) Image of a fibre–microlens probe with super continuum laser coupled in. (vi) A scanning optical microscopy experiment shown schematically. (vii) Reconstruction of the excitation electric field intensity in the *xz* plane, showing the Gaussian distribution fitted to the *xy* intensity at each *z*′ position. (viii) Reconstruction of the excitation field intensity in the *yz* plane, showing the fitted Gaussian at each *z*′ position. (iv) Measured peak intensity at the centre of the Gaussian fit (*x*_0_, *y*_0_) at each *z*′ position. Adapted/reproduced from ref. 19 with permission from American Chemical Society under Creative Commons CC-BY-4.0, Groom *et al. ACS Photonics*, 2024, **11**, 3167–3177, copyright 2024.

A notable accessory is a probe element, for immersive (liquid) monitoring, perhaps disposable, which can be used for easy real-time process monitoring, and may also involve flow cells in the case of the assay being brought to the system^15^ (Fig. 4). If not immersive, such probes typically have a short working distance to the sample, say within 100 mm, allowing almost a point-and-shoot operation. Some probe elements may mean a reduced spectral range, which may preclude analysis of high-wavenumber Raman bands (*i.e.* greater energy change photons). The loss of higher wavenumber Raman signal might be unfortunate, having many useful diagnostic purposes including tracking changes in adipose tissue.^16^*In vivo* use probes present further challenges: they must be small enough for body analysis, non-toxic and sterilisable.^17^ Whereas on-skin analyses and cm-scale diameter probes have similar performance to microscope-based systems, this is less so as probe diameter is miniaturised for *in vivo* interrogations. The engineering challenges associated with endoscopic probes are significant, especially for solid organs *in vivo*, which need sub-mm diameter probe sizes. For instance, in a gastro-endoscopic context, probes are required to be smaller than 2.8 mm diameter. In a special case, Mowbray *et al.* list a larger Raman probe inner diameter of 14 mm in a study into deploying a Raman probe element through a cranial burr hole for traumatic brain injury assessment.^18^ Assimilating miniatured lenses and filters in such devices is challenging and their performance must be carefully characterised, for example, as in the 28 μm barium titanate glass microlens embedded in the hollow-core fibre in the study of Groom *et al.*^19^ A fibre-optic medium can be thought of as a ‘large’ waveguide, where a high refractive index core, typically (fluorine, boron, titanium-doped) silica (SiO_2_), and lower refractive index encapsulating cladding, causing total internal reflection.^17^ Typically, the thinnest silica fibres are about 500 μm with a spatial resolution of hundreds μm. Notably, Yamanaka (2016) developed a hair-like fibre probe of two 30 μm diameter silica fibres for *in vivo* examination,^20^ with 23 μm resolution and which confers an additional benefit of fluorescence rejection, key for low background in Raman signals,^21^ usually tackled by confocal arrangements,^22^ careful selection of laser wavelengths, or algorithmic post-processing for background removal. The authors in ref. 20 postulate that this effect of lower background fluorescence is as a result of is the suppression of secondary fluorescence event capture, stemming from the probe geometry.

The utility of fibre-probe based Raman instruments is acutely dependent on application. For instance, while a spatially imprecise measurement may suffice for a homogeneous sample measurement, compositionally heterogeneous samples may require high spatial resolution Raman maps.^23^ One example is in breast cancer. While hand-held probes have been used to identify positive cancer margins, *e.g.* brain cancer^24^ and cervical cancers,^25^ specific cancers may prove less amenable to hand-held probe analysis; for instance, ductal carcinoma *in situ* (DCSI), which is responsible for high rates of re-excision in breast cancer.^26^ Here, a microscopy/high-resolution Raman mapping approach is required for accurate pathological identification.^23^ Elsewhere, some portable instrumentation may require outside use, and the variation in ambient conditions can cause problems like detector saturation or variable background, which are not present (or easily controlled) in lab-based instrument use.^27^ Although a laser spot size on order of 10 μm is possible, the actual location measured will be limited by ‘handshake’ by the operator, perhaps as much as 2 mm, which may or may not be sufficient depending on the nature of sample inhomogeneity and desire to measure specific regions of compositional interest. At best, human hand tremor is at least at the level on the order of 100 μm, and thus precise robotic, or co-operative robotic, control may still be required. Microscope attachments add undoubted complexity to portable devices and still may not provide needed locational accuracy in some cases.^27^

## 3. Laboratory instruments to handheld devices

Essentially all elements of Raman systems can be downsized. Nevertheless, small forms are limiting for spectral resolution and range, and this can reduce the utility of Raman spectroscopy at the smallest scales, which is normally characterised by inherently sharp peaks, and thus high analytical specificity and multiplexing capabilities.^21^

Full integration of all sensor components, *i.e.* light source, detector, sample analysis region, is costly, chiefly due to alignment and in- and out coupling of light.^29^ Thus, small, yet ‘separated’ or ‘modular’ Raman systems are attractive. Sharma and colleagues have examined a range of miniature heterodyne (*i.e.* signal frequency mixing) Raman spectrometer systems, which, like Fourier transform Raman analyse spectra in the time domain but without moving parts *i.e.* mirrors, required in a normal interferometric set-up. The system further bypasses the throughput limitation imposed by the finite-size slit aperture in a conventional dispersive Raman spectrometer.^30^ The group have explored one-grating^31^ and two-grating systems (with integrated beamsplitter) of varying size,^32^ monolithically, or fabricated in multiple parts, most recently as light as 60 g with a footprint as small as 6 cm^3^ and 8 cm^−1^ spectral resolution,^30^ which is competitive for a miniature system. These small Raman systems are designed for external light sources and sensing elements, a simpler arrangement than on-chip solutions.

Despite different sizes and arrangements, most of the components of Raman systems remain the same, with some exceptions, where different parts or interchangeability is needed. This often corresponds to special kinds of experiments. For example, multiwavelength SERS studies – surface enhanced Raman excitation spectroscopy (SERES) – will need a mechanism to exchange the laser line (bandpass) optical filters and Rayleigh (longpass) filters depending on the laser wavelength used. Consequently, SERES typically remains a research tool confined to large benchtop Raman systems. Generally speaking, the optimal laser wavelength(s) needed is dependent on applications and the exact type of Raman experiment – corresponding to resonance wavelengths for the target molecules, fluorescence rejection, or wavelength for plasmon resonance in SERS.^21^ In another kind of Raman experiment, notch filters can be used in place of normal Rayleigh cut-off optical filters where anti-Stokes Raman bands (*i.e.* energy transfer from molecule to Raman photons) are required, which are typically much weaker but useful for sample temperature determination when compared to the Stokes Raman band intensities. THz (low-frequency Raman) requires especially narrow cut-offs (*e.g.*, within tens cm^−1^) to capture the array of Raman bands associated with structural information in this range. For instance, the authors in ref. 31 use two different Rayleigh filters because of overlapping wavelengths above and below the grating blaze wavelength *i.e.* same spatial frequency (Fizeau fringes) in their diffraction grating-based interferometric Raman system. Elsewhere, systems for measuring Raman at a depth, spatially offset Raman spectroscopy (SORS), require an offset detection element to monitor backscattered photons from within a sample.^33–35^

Even with technological advances, many trade-offs in performance exist as devices shrink; for example, one notable limitation of miniaturised Raman systems is laser stabilisation. Small form factor Raman systems typically use diode lasers, which are cheaper than HeNe or solid state lasers, but with a trade off in performance in terms of wavelength (spectral) and power output stability. Ilchenko (2024) proposed a cm-scale miniaturised Raman spectrometer composed of non-stabilised laser diodes, densely packed optics, and non-cooled small sensors.^36^ The authors achieved excellent sensitivity comparable to bulky research-grade Raman systems, and an overall 7 cm^−1^ resolution within the fingerprint region by using an in-built calibration material. Recently, we have provided a summary of the spectral resolutions in commonly available benchtop and handheld Raman systems, which spanned from 1 cm^−1^ to 11 cm^−1^. A spatially offset Raman device with 830 nm excitation laser had a spectral resolution of 14 cm^−1^ (see SI section ref. 21). These values are dependent on the laser wavelength and exact part of the spectrum analysed (different Raman peaks are at different Raman-scattered wavelengths). Often, a vendor will provide a range of values. Further integration/miniaturisation of Raman devices will push these values 10 cm^−1^+ and have a greater reliance on algorithmic means in interpreting spectra. The usefulness of this will then depend on application, and this may mean that on-chip Raman devices, if they are available routinely as a commercial product, may be very application-specific.

### 3.1. Spectrometer miniaturisation

Of particular note in efforts on Raman system downsizing is smaller spectrometer development,^37,38^ as the spectrometer is a critical component of any Raman system, splitting the Raman-scattered light up so that the relevant part of the spectrum can be analysed. By 2030, the spectrometer market is projected to pass 4b USD, a 9% compound annual growth rate (CAGR) from 2021–2030, dominated by optical and mass spectrometry technologies across life sciences, chemical, and food/agriculture applications.^39^ Ref. 40 includes ‘geology, mining and oil and gas’ alongside forensics as notable application spaces for ‘compact’ spectrometer systems. Further, a significant proportion of this could be labelled ‘mini- or micro-spectrometers’, those fitting into the palm of your hand, which commanded a 900m USD market share based on a 2020 report.^41^

Predictably, downscaling spectrometers comes at a performance trade-off cost in terms of measurement speed, spectral bandwidth, resolution, and dynamic range, and hence must be tailored to the specific application. Cooling strategies must normally be found where thermal management may be difficult on the compact scale. Integration with prevalent CMOS silicon fabrication and use of heterogeneous integration for on-chip lasers and detectors is one strategy to keep costs down.^42^ Specifically, maintaining etendue (collected light solid angle × sample collection area) conservation of the optical set-up is to be considered. Here, then, the bottleneck is the least effective component.^17^

In an assessment of the current state-of-play of portable spectroscopy, Crocombe defines four different categories of mini-spectrometer use:^43^

1. Stand-alone spectrometers for laboratory use,

2. Integrated spectrometers in portable spectroscopic devices,

3. Small spectrometers in consumer goods, and

4. Spectrometers marketed directly to consumers.

Category 4 is an especially interesting suggestion, but one which may find regulatory pushback. A 2021 report suggests market penetration for consumer applications as minor^40^ but this is expected to steadily increase.^41^ Crocombe highlights white goods and consumer health and care products but interest in on-chip Raman could mean applications could conceivably go much further.^43^ Recently Li *et al.* (2022) has provided a useful review on the current standing of miniaturised spectrometers.^42^ Such systems will still require small parts, and to achieve increasingly compact components in Raman systems entails a focus on reliable manufacture, spurred by recent developments in lithography.^8^

## 4. MEMS-based components in Raman miniaturisation

**Fig. 5:**
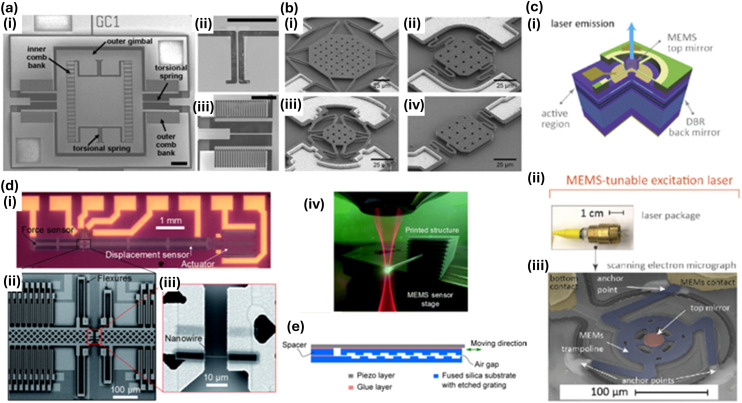
Types of MEMS devices and technologies used in miniaturisation of Raman and other systems. (a) (i) Scanning electron microscopy (SEM) images of a two-dimensional MEMS scanning mirror, (ii) inner axis torsional spring, (iii) outer axis comb bank. Scale bars are 250 μm. Adapted/reproduced from ref. 61 with permission from Optica Publishing Group, Piyawattanametha *et al. Opt. Lett.*, 2006, **31**, 2018–2020, copyright 2006. (b) (i–iv) SEM images of polycrystalline diamond hotplates. Adapted/reproduced from ref. 71 with permission from John Wiley & Sons under Creative Commons CC-BY-3.0. Thomas *et al. Small*, 2023, **19**, 2303976, copyright 2023. (c) (i) Schematic of a chip-scale MEMS-tuneable vertical cavity surface emitting laser (VCSEL), (ii) and (iii) show the photo and SEM image of a MEMS-tuneable VCSEL. Adapted/reproduced from ref. 47 with permission from Optica Publishing Group Atabaki *et al.*, *Opt. Express*, 2021, **29**, 24723–24734, copyright 2021. (d) MEMS-based miniaturised tensile tester, (i) optical image of the tensile tester, (ii) and (iii) show the SEM images of the contact pads and the polymer nanowire, (iv) schematic of printing a nanowire on top of a MEMS sensor. Adapted/reproduced from ref. 66 with permission from Royal Society of Chemistry under Creative Commons CC-BY-3.0, Ladner *et al.*, *RSC Adv.*, 2019. **9**, 28808–28813, copyright 2019. (e) Schematic of a MEMS-based tuneable grating with integrated actuation. Adapted/reproduced from ref. 57 with permission from Optica Publishing Group, Stürmer *et al.*, 2016, *Opt. Exp.*, **24**, 23765–23776, copyright 2016.

Micro-electro-mechanical systems (MEMS) is a crucial technology in the miniaturisation of various devices, including Raman spectroscopy, referring to micro-scale systems (1–100 μm) involving electronic and moving components. MEMS includes sensors, actuators, and electronics on a common substrate using microfabrication technologies and their integration.^44^ These systems offer several advantages, including miniaturisation and low power consumption, making them ideal for portable and handheld devices. In recent times, MEMS has significantly aided the miniaturisation of Raman spectroscopy devices to make them portable and efficient analytical systems (Fig. 5). An overview of MEMS-based items in Raman spectroscopy is below.

### 4.1. Laser sources

MEMS-based laser sources have been used in the development of compact Raman spectrometers. Tuneable MEMS-based vertical cavity surface-emitting lasers (VCSELs), which are compact, efficient, and capable of precise wavelength tuning, are thereby suitable for portable applications in Raman systems.^45,46^ While traditional VCSELs are not MEMS technology based (no moving parts) MEMS-VCSELS have a moving distributed Bragg reflector (DBR) layer (usually electrostatic activation) to tune the emission wavelength. The wavelength tuning range is typically nms to tens nm in the near-infrared range. MEMS laser sources enable the easy selection of various laser wavelengths,^47^ which is essential for applications requiring multiple excitation wavelengths,^21^ for instance, a device to target a wide range of molecules, resonant at different wavelengths, perhaps even a portable multiwavelength SERS system. In addition, multiple clearly spaced wavelengths are key to sequentially enhanced Raman difference spectroscopy (SERDS), which can then be used to effectively subtract the fluorescence background, which remains spectrally fixed. Most MEMS VCSEL uses, however, remain in the IR. The key problem with visible-range VCSEL use is that more DBR layers are required with a strong lattice mismatch for a suitable laser. Fabrication becomes increasingly difficult beyond the red region into the green and blue visible regions.

Hayden *et al.* used MEMS-based VCSELs for wavelength modulation spectroscopy to demonstrate a CO_2_ detection limit 2.5× lower than previous measurements.^46^ Pei *et al.* demonstrated a near-single-mode fibre Raman laser system with a power of 7.11 W at 1560 nm band.^48^ Additionally, MEMS-based laser diodes have been used for high-power and low-divergence beams that are required for deep tissue penetration in biomedical applications.^49^

### 4.2. Optical filters

Optical filters are essential for the selection of specific wavelengths in Raman spectrometers. Miniaturisation of multilayered dielectric stacks, as in conventional optical filters, is problematic because their function is highly angle-dependent. Small form factor Raman systems, having a fixed focal length, need lenses with larger numerical apertures to gather as much of the (low number of) Raman photons as possible, inducing less uniform light *i.e.* aberrations (coma, astigmatism). High-angled incident light can also result in (unwanted) polarisation-selective transmission. Further, heat control often becomes tricky at this scale and can affect refractive indices and thus cut-off wavelengths. Alternatively, MEMS-based filters such as Fabry–Perot interferometers significantly assist in the miniaturisation of Raman systems by bypassing some of these problems and offering high spectral resolution, low power consumption and dynamic tunability, making them ideal for portable and point-of-care applications.^50–53^ They have been used in point-of-care diagnostics and environmental monitoring, where compact and robust systems are essential. Russin *et al.* developed a MEMS-based Fabry–Perot interferometer (FPI) for integration into a Raman system achieving a reflectivity of 97.3% at 1550 nm.^51^ The interferometer had a natural spectral linewidth of 70 cm^−1^ and a free spectral range (FSR) of 334 cm^−1^ – the spectral range that a spectrometer can analyse without interference from higher orders of diffraction. Recent advancements include hybrid MEMS filters capable of operating across multiple wavelength ranges, enhancing the adaptability of Raman devices for diverse applications.^54,55^ The problems with FPIs in Raman include poor optical density (OD) rejection at the Raman laser wavelength and the periodicity of the transmitted light (multiple FPIs may be required). Moreover, the angle sensitivity is, in fact, worse than with edge filters. This means that unless device compactness and tunability, and perhaps electronic calibration, are paramount, conventional optical filters remain best (mm-sized and larger) despite fabrication challenges for these optical elements at this scale.

### 4.3. Gratings and spectrometers

MEMS gratings and spectrometers are essential components for analysing the Raman spectrum as they enable the precise detection and separation of the weak Raman-shifted light from a sample.^56,57^ MEMS-based diffraction gratings and on-chip Fourier transform spectrometers (FTS) enable high-resolution spectral analysis in a compact form. For instance, a multi-aperture silicon nitride waveguide-based FTS achieved a spectral range of 40 nm and a resolution of 0.5 nm.^58^ Recent advancements include the development of ultra-compact MEMS-based spectrometers that achieve sub-nanometre resolution, suitable for portable Raman applications. Ismail *et al.* developed a wavelength selective arrayed-waveguide grating using silicon oxynitride technology.^59^ The central wavelength was 901 nm with a FSR of ∼22 nm and a resolution of 0.2 nm. This kind of FSR-resolution balance is on the comparatively high resolution – narrow spectral range (about a 800 cm^−1^ or 400 cm^−1^ span at 780 nm or 532 nm laser excitations, respectively), and thus probably interesting for targeted *i.e.* application-specific detections (rather than broad application use). Kerber *et al.* designed subwavelength gratings for incident wavelength of 1500 nm, offering increased reflectance over dielectric mirrors.^60^

### 4.4. Mirrors

MEMS mirrors are used in Raman systems for beam steering and scanning. Dual-axis MEMS scanning mirrors provide precise control over the laser beam path and provide improved spatial resolution.^61^ These mirrors are especially critical in confocal Raman systems. Zhang *et al.* developed a MEMS-based Raman probe for geological exploration using dual 2D MEMS mirrors to achieve a lateral resolution of 500 nm and axial focusing capability of 50 nm.^62^ This method used the two MEMS mirrors for beam telecentric scanning (where the chief ray stays parallel to the optical axis ensuring the angle of incidence, magnification and light spot size stay the same over the scanned area), eliminating the traditional relay system, significantly compressing the size of the confocal imaging probe, and improving the detection efficiency of Raman signal. The planar reflective structure avoids the introduction of aberrations. Moreover, this method used Rayleigh light and reflected light to construct a real-time focus tracking ability system, which improves the spatial resolution and anti-drift ability of the confocal Raman detection system, and can achieve simultaneous rapid topographic and Raman mapping with high resolution. Elsewhere, a rapid imaging method has been developed by combining dual MEMS mirror and grid-by-grid scanning methods,^63^ achieving an imaging speed 45 times higher than traditional point-scan confocal Raman systems. Recent advances include the development of MEMS mirrors with integrated feedback control systems for dynamic focusing and stabilisation, enhancing the reliability of portable Raman systems in dynamic environments.^64^

### 4.5. Photodetectors

Photodetectors are designed to work with MEMS gratings and filters, enabling real-time data acquisition and analysis. Advances in MEMS detectors have made Raman systems more efficient and capable of detecting weak signals in compact devices. The inherently weak Raman scattering signal makes this a critical consideration, alongside stray light rejection and potential means of signal enhancement, like SERS. MEMS-based photodetectors can operate across a wide spectral range, from visible to infrared. These detectors have been used in Raman systems to enhance signal detection in low-light conditions, particularly for biological and forensic applications. Grotevent *et al.* demonstrated a metal-oxide semiconductor-based colloidal quantum dot photodetector in an ultracompact FTS, thereby exhibiting a spectral resolution of 50 cm^−1^ at an active spectrometer volume below 100 μm × 100 μm × 100 μm.^65^ MEMS detector arrays have been developed that offer multi-wavelength capabilities, reducing the need for separate components and improving the compactness of Raman systems.

### 4.6. Actuators

MEMS actuators facilitate precise movement and positioning of optical components within Raman systems.^66^ These actuators are essential for maintaining optimal system performance in varying environmental conditions. MEMS-based thermal actuators and comb-drive actuators are commonly used in Raman systems for fine-tuning and alignment. MEMS actuators have been used to adjust the alignment of Fabry–Perot cavities and gratings in portable Raman devices. Lavrik *et al.* developed microcantilever-based actuators to integrate chemi-mechanical transduction and SERS.^54^ Noble metal nanoparticles were deposited on the microcantilevers and strategically used as sensor–actuators to generate substantial SERS signal. Xie *et al.* developed voltage-controlled MEMS-based comb-drive actuators for broad frequency tuning.^67^ By controlling strain using actuation voltage, the authors tuned the continuous resonance frequency up to 75%. Yao *et al.* developed a microheater-integrated microcantilever and combined it with a Raman system and conducted simultaneous thermogravimetric and Raman analyses using a heating process.^68^

### 4.7. MEMS microheaters

MEMS-based microheaters can be employed in Raman systems, especially in gas sensing applications.^69^ These heaters provide precise temperature control, ensuring consistent Raman signal quality. A MEMS-based hydrogen gas sensor with a platinum micro-heater demonstrated a response time of 39 seconds and a recovery time of 35 seconds while consuming just 2.19 mW of power.^70^ Another notable application is in temperature-programmed Raman spectroscopy, where MEMS micro-heaters are used to control the temperature of samples during Raman measurements.^68^ This approach allows researchers to study phase transitions and chemical reactions in real-time. Such temperature control can be used in conjunction with calculations of the local temperature at the point of measurement which uses the relative Stokes (energy increase), anti-Stokes (energy decrease) Raman peak intensities.

Micro-heaters are also used to prevent condensation and stabilise the sample environment during Raman measurements, improving the reliability of portable Raman devices. Advanced MEMS micro-heaters made from boron-doped polycrystalline diamond can reach temperatures of up to 2731 K with minimal power consumption, making them suitable for various industrial and environmental monitoring applications.^71^ These micro-heating systems enhance the accuracy and reproducibility of Raman measurements. Aside from the above applications, we note the possible use of MEMS-based microheaters in the form of (thermal) sequentially shifted excitation (SSE) Raman spectroscopy^72,73^ to induce slight temperature changes in the sample, which can permit the effective removal of fluorescent background, analogous to SERDS, that does not spectrally shift as temperature changes, as the Raman peaks do.

### 4.8. MEMS integration with photonic platforms

Recent advances have focused on integrating MEMS-tunable lasers with photonic integrated circuits (PICs) to achieve fully miniaturised Raman systems. These platforms combine MEMS actuators with silicon or silicon nitride waveguides, enabling on-chip wavelength tuning, coupling, and filtering without bulky external optics. For example, silicon photonic MEMS allow wafer-scale integration of tunable VCSELs alongside optical couplers and phase shifters, creating compact, robust Raman modules suitable for field deployment.^74^

Hybrid integration of III–V MEMS lasers is another emerging area *i.e.* using compound semiconductor materials from group III, IV, and V materials like GaAs, InP, InGaAsP, AlGaAs with direct bandgaps and thus high light emissions. This hydrised integration with silicon PICs has produced mode-hop-free tunable lasers with narrow linewidths (<6 kHz) and wide tuning ranges (>20 nm), ideal for Raman and atomic spectroscopy applications.^75^ Similarly, waveguide-enhanced Raman spectroscopy (WERS) on nanophotonic chips can leverage MEMS-integrated sources to excite analytes directly within low-loss waveguides, improving sensitivity while maintaining a small footprint^76^ – a kind of on-chip Raman. These developments pave the way for next-generation fully chip-scale mini-Raman systems, combining MEMS tunable lasers, integrated optics, and detectors.

## 5. On-chip Raman

**Fig. 6:**
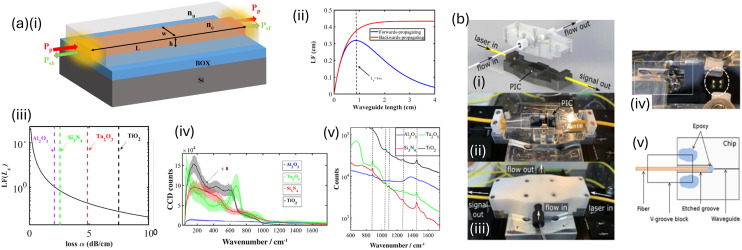
On-chip/waveguide Raman. (a) ‘High index contrast photonic platforms for on-chip Raman spectroscopy.’ (i) The schematic of a patterned high index contrast waveguide. *n*_c_ and *n*_a_ are the refractive index of core and analyte (top cladding) respectively. (ii) The length factor (LF) comparison for a forward (blue) and a backward (red) propagating Raman signal assuming a fixed waveguide loss of = 5 dB cm^−1^. (iii) The LF increases for decreasing loss (see eqn (1) in ref. 80, Raza *et al.*). The dotted lines show the value of the LF at their saturation length (1/waveguide loss) for the four different types of waveguides. (iv) Background scattering from four different waveguide platforms normalized by length factor and coupling efficiency. (v) The Raman spectra of ethanol at the upper cladding are obtained. The dotted lines represent the 880-, 1054-, 1098-, 1275-, and 1456 cm^−1^ Raman modes of ethanol. Adapted/reproduced from ref. 80 with permission from Optica Publishing Group under OSA Open Access Publishing Agreement, Raza *et al.*, *Opt. Exp.*, 2019, **27**, 23067–23079, copyright 2019. (b) ‘A packaged, fibre-coupled waveguide-enhanced Raman spectroscopic sensor’. (i) Rendering of the chip enclosure with optical fibres affixed to the chip and a flowcell that lowers onto the surface of the chip. The top case and flow channels are machined from polytetrafluoroethylene (PTFE), a rubber Kalrez O-ring separates the chip from the top case, and the bottom fixture is machined from aluminium. (ii) Photograph of the fibre-coupled, packaged Raman sensing chip with fibres glued to the edge of the chip after being mounted in separate glass V-groove chips (for mounting purposes). (iii) The packaged chip with the top PTFE cell secured above the chip. (iv) Zoomed-in top view of the area boxed in white on (ii). The position of the O-ring is shown by the dashed white circle (the side clamps are removed prior to O-ring positioning). (v) Top-view schematics of the fibre-bonding region [boxed in white on (iv)]. Components are not to scale. The glass V-groove block is bonded to the chip with epoxy at the tip of the fibre in the etched groove, and with larger volumes of cured epoxy on the sides of the block for improved robustness. Adapted/reproduced from ref. 82 with permission from Optica Publishing Group, Kita *et al.*, *Opt. Exp.*, 2020, **28**, 14963, copyright 2020.

What is ‘on-chip Raman spectroscopy’? (Fig. 6) The term is often seen as synonymous with ‘waveguide Raman’ which is a highly integrated miniaturised device without many of the optical components usually used in Raman spectroscopy,^37,77–82^ also often incorporating the use of SERS-based assays^83–85^ (see section 6) and microfluidics^86–89^ (see section 7.1.). Waveguide Raman systems, specifically, refer to those on-chip Raman systems that use a guided optical mode of light to do Raman sensing – ‘on-chip’ is a broader term encompassing any small Raman device *e.g.* plasmonic nanostructures for SERS. Notably, fibre optic interconnects, for example, can be replaced by silicon waveguiding. The greater material contrast with the surrounding medium means smaller bending radii are possible, down to the μm-scale.^28^ Although illumination and detection are typically integrated, a fully self-contained Raman system is not always implied. Sample injection should be straightforward and microfluidic incorporation preferable.^90^ Counterintuitively, such integrated systems need not necessarily be multi-use, as low-cost manufacture, even of components such as microscale lasers, means the chips could be disposable.^28^ For mass testing applications, as with the recent COVID-19 lateral flow assays, cheap, low sensitivity, disposable sensors may be preferable. Otherwise, sensor amenability to reuse needs to be carefully considered. In calibration experiments in ref. 90, for instance, a Raman quartz waveguide chip was purged with 250 μL of distilled water between measurements. Reusability and memory effects can prove problematic as noted with nanoparticle fouling in the SERS studies in ref. 91–93.

The waveguide channel serves two purposes – 1. as an effective medium to facilitate interaction with proximal analyte molecules (surrounding the core) and 2. an easy way to collect the Raman signal (with appropriate filtering of the Rayleigh signal).^78,94^ One of the problems with on-chip Raman is that waveguides are typically single-mode, meaning that they only efficiently propagate a certain profile of light travelling in the confined space. This is because waveguides are designed to be compatible with wavelength-specific components. On the other hand, Raman emission is inherently multi-mode light, originating from a diffuse source of analyte molecules and thus coupling efficiency is low, compounding already an inherently low signal (cross-section) from the Raman scattering phenomenon. The waveguide can be considered the functional component of an on-chip system where the Raman interaction occurs; however, the wider chip can also be largely photonic-based, aligning with efforts to develop fully light-based miniature systems to replace electronic infrastructure.

### 5.1. System electronics and photonic integrated circuits

A crucial aspect of a compact Raman spectroscopy system are the electronics, which connect the detector signal to a visible output for the user. Efforts to minimise computational and electronic components have, by now, a long history. 1950s computers were spacious; for instance, the first computer with magnetic storage media, RAMAC, in 1955 could fill a small room. Initially, incandescent vacuum tubes, which could rapidly switch on to off about 10 000 times per second, were used as electronic switches, later replaced by small semiconductor devices, transistors. At the time, this was revolutionary, vacuum tubes had short lifetimes and the light could attract moths, meaning one's computer might need ‘debugging’. Transistors, however, did not solve all problems because small devices required many interconnects, which was not feasible until the advent of the microchip.^96^

Developments in complementary metal oxide semiconductor (CMOS) technology in the early 90s meant that photodetectors, readout circuits, and data processing units could be placed on the same chip. The CMOS active pixel sensor architecture significantly increased readout speed, reduced size and lowered power needs.^97^ Fabrication improvements in the 2000s resulted in more complex analogue circuits *e.g.* analogue to digital converters, and rudimentary digital circuits on the same chip.^9^ More recently, light-based circuit technology has garnered interest – photonic integrated circuits (PICs) – which can replace electrons with light and can lower costs and size in optical sensing devices, having been proposed for medical wearable technology, which for example can monitor blood sugar or alcohol levels.^98^ Such examples are truly system-on-chip (SoC) designs that incorporate data acquisition, processing and communication into a single chip.^9^ While typically much larger than electronics, μms *vs.* nms say, PICs are starting to garner interest as an alternative technology. The real driver for the adoption of PICs is a reduction in losses, while being much more lossy than optical fibres, have significantly lower losses than electrical circuits.

PICs are also a promising vehicle for miniaturised sensing, allowing the use of photons as part of the device to investigate molecules of interest. While unlikely to fully replace silicon electronics, PICs open up new possibilities including easy multiplexed analyses of complex matrices.^29^ Many sensing PIC systems are currently surface plasmon resonance-based, for example in a micro-ring resonator or Mach–Zehnder interferometry arrangement, but on-chip Raman systems have appeared for creatine detection in the context of kidney disease and sarin (nerve) gas.^29^ PIC material choice is important. Si photonics refers to processes used to make PICs using existing CMOS processes. Silicon, however, is only transparent above 1.1 μm but silicon nitride can be used (Si_3_N_4_) for transparency in the visible range, needed by Raman and fluorescence.^94^ Silicon nitride thus offers a less lossy option than silicon and is ideal for the visible and near infrared range, having a wider bandgap. Further, the high refractive index contrast of silicon nitride aids electric field concentration.^94^ Indium phosphide (InP) offers an alternative for active components – light sources and detectors. Hybrid integration (different chips packaged together *via* lateral edge coupling or vertical flip chip) and heterogeneous integration (different materials) are now achievable and scalable.

### 5.2. Alternative arrangements

More exotic arrangements are possible in fully miniaturised Raman systems. The authors in ref. 90 employ a photonic trap to maintain the position of their target, polystyrene spheres. Optical traps have promise in Raman for manipulation on the nanoscale, permitting single cell placement and analysis,^99,100^ and previously optical forces have been identified as potentially very useful in nanomanipulation in plasmonic systems, perhaps forming ‘optical factories’.^101^ Elsewhere, dielectrophoretic^102^ (forces acting on particles due to polarisation in non-uniform electric fields) and magnetic effects^103^ have also been useful in Raman experiments to manipulate plasmonic media and could be incorporated in smaller on-chip systems. An array of functionalised Raman arrangements exist,^2^ including highly selective molecular imprinting techniques^104^ (like a highly selective molecular ‘lock-and-key’), and could find use in disposable Raman chips, combined with data analysis strategies to extract information on subtle spectral changes corresponding to binding events.^105^ Another interesting aspect is the use of quantum dots, sub-10 nm particles of semiconductor, of varying size, that can efficiently absorb and emit light due to unique quantum confinement effects, replacing gratings and filters and can be used for multispectral implementations.^9^ Elsewhere there have been foci on low-cost on-chip spectrometers, based on Echelle gratings, arrayed waveguide gratings and planar concave gratings, which use visible and near-infrared light sources.^106–108^ The drawback of such technology lies in poor signal-to-noise ratio (SNR) due to the input light being distributed over many spectral channels.

Wuytens *et al.* developed intracellular Raman probes using gold nanoparticles for enhancement of Si_3_N_4_ with a chip size on the μm-scale.^109^ The same researchers have also patterned nanostructures within waveguides for increased Raman signal *via* similar plasmonic enhancement. The authors have fabricated bowties nanostructures, which have received extensive research interest in SERS and wider plasmonics research as a high sensitivity nanoplatform with a geometry amenable to resonance wavelength tuning for optimum concentration of impinging light at a wavelength of choice.^110^

### 5.3. Background signals in on-chip Raman systems

Finally, background signals can prove problematic, often swamping the (comparatively low) Raman signal. This includes shot noise, arising from the quantum nature of electric charge, background signal associated with the waveguide material,^95^ or a Raman signature that interferes with Raman peaks of the analyte either from the base material^111^ or interconnects. Similarly, a low Raman signal might be problematic for less sensitive detectors and may benefit from a waveguide material with a lower dark current *i.e.* InP. Meanwhile, the relationship between Raman collection efficiency and index contrast has been shown to be quadratic in nature.^112^ Si_3_N_4_, for example, which can offer greater transparency in the visible wavelength range amenable to the analysis of biological samples, could be useful,^95^ retaining index contrast with the surrounding environment for efficient collection of the Raman signal. The effect of waveguide crystallinity may also play a role where background signal may be enhanced or reduced at some wavelengths *via* interaction, or lack thereof, with phonons.^113^

Dochow *et al.* developed hexagonally arranged, multicore (*n* = 61) single-mode optical fibres (NA = 0.1; *d* = 5.5 μm) with UV-patterned fibre Bragg gratings close to the Raman Stokes light pick-up point to suppress background from the optical fibres in an on-chip Raman device.^90^ Use of filters can also be reduced by using high wavenumbers where silica has no interfering Raman response. Similarly, autofluorescence from silicon dioxide cladding can be a problem.^17,94^ In the context of reducing the impact of background signal, Le Thomas *et al.* have discussed different strategies. Clear approaches exist involving analyte enrichment with an absorbing/adsorbing layer to concentrate target molecules, or employing resonant Raman scattering where the laser wavelength corresponds to a molecular resonance and thus returns a larger Raman signal, perhaps up to 10^3^×. This, then, entails also using a waveguide medium suitable at this resonant wavelength – the authors have recently studied aluminium oxide and 360 nm excitation.

Away from material considerations, advanced data analysis strategies can also mitigate the effect of background signals in on-chip devices. For example, in a study into cancerous cell identification, Dochow *et al.* employ a statistics-sensitive non-linear iterative peak-clipping (SNIP) algorithm for background identification and the commonly used, dimension-reducing, principal component analysis (PCA) process, but for noise removal, in combination with optical trapping within a microfluidic Raman device.^114^ Further, the authors use normalisation to a prominent high-wavenumber C–H stretch Raman peak, alongside spectral truncation. We note that the exact algorithms used and indeed the precise order in which these various processes are performed could be critical, as discussed in a Raman review on saliva as a diagnostic biofluid.^14^ An optimistic view on dealing with background, pertaining mostly to fluorescence from Raman dye molecules, say, should be positive as algorithms for background subtraction have improved in recent years. However, spectral processing from portable devices, and the resultant spectra, will still need frequent checking by human actors, arguably like a lot of other uses of advanced data analysis applications. Another option to limit the effect of background influence is to increase signal *via* the incorporation of plasmonic enhancing media – surface enhanced Raman spectroscopy.

## 6. Incorporation of SERS-enhancing media

Surface enhanced Raman spectroscopy (SERS) is a surface-sensitive technique that enhances the Raman signal predominantly by large electromagnetic (EM) fields that arise around metal-coated nanostructures,^115^ which can couple to the incident (laser) and outgoing (then Raman-scattered) photons. The intense local EM fields are caused by the excitation of surface plasmon-polaritons, hybrid electron-light excitations propagating at a metal–dielectric interface. Metals such as gold and silver support these surface excitations and are commonly employed. Depending on how the Raman enhancement is calculated exactly,^116^ this increase in the Raman signal can be as large as 10^12^ for specific locations.^117^ An enhancement factor of 10^4^–10^6^ is more applicable for a substrate-averaged calculation (a more useful metric).^117^

SERS substrates are generally viewed as consumable. This has proved problematic for precisely engineered SERS architectures, say *via* electron beam lithography, which then have a prohibitively high cost per measurement. Reusability in SERS substrates have been explored but with little traction. Thus, low-cost, less reproducible nanoparticles, which retain a high enhancement and whose properties are well-understood, have remained popular. Research into ordered SERS substrates is ongoing, attracting each year, hundreds of publications, offering better spot-to-spot uniformity of signal and batch-to-batch reproducibility than nanoparticle counterparts.

At the time of writing the authors' opinion is that future SERS chips will probably continue to form as accessories to Raman systems, rather than in-built components. This is for a number of reasons:

1. This allows the spectroscopist the option to perform ‘normal’ Raman spectroscopy, which may be adequate in the high concentration regime, without having to consider the effects of adsorbing to a plasmonic surface.

2. Unnecessary device complexity and economy of materials – simpler Raman devices (hand-held or chip-scale), and without the need for additional cost of gold/silver nano-structures – although we note that while top-down fabricated SERS substrates *e.g.* electron beam lithography, have proved prohibitively expensive, the cost of metal nanoparticles/colloids is very cheap. The cost per measurement will be critical to SERS full adoption by the analytical community, which, needs to be cents rather than dollars or even $10s (as is often the case).

3. No concerns of device shelf-life – incorporation of plasmonic nanostructures for SERS brings with it concerns over device shelf-life, especially true for silver-based nanostructures, which oxidise rapidly. Having the SERS chip externally packed, which can be opened and then measured by a hand-held Raman system, or alternatively, say, nanoparticles dropcasted or flowed through an on-chip Raman microfluidic system, bypasses such concerns.

4. Reusability for on-chip devices – SERS is normally considered a single measurement technique. Even where molecules can be removed from a surface, or a microfluidic channel exists, some residue, analyte molecules or nanoparticle fouling often remains. The upshot being a less cost-effective device.

In addition to the points noted above, is the contention that SERS cannot be a fully analytical technique. For instance, a histogram of SERS enhancements across a SERS-active area often shows skewed ‘long tail’ statistics, *i.e.* high kurtosis, meaning a small number of surface sites on the SERS-active area contribute disproportionately to the observed SERS signal.^118^ This is problematic then from a reproducibility and quantification perspective – the number of these ‘hot hotspots’ can vary from batch to batch. Thus, many see SERS as still in need of development in order to make an application impact,^117,119^ and having not met its potential as an analytical technique. We understand these concerns, and are sympathetic to them, realising that the electromagnetic hotspots that can be formed *via* plasmon interactions in the nano-geometries mediating SERS can be difficult to control. However, we would argue that SERS might be like any other analytical technique in that it can avail from calibration *e.g.* by an internal standard. Elsewhere, focus has been given to better fabrication strategies as noted by Dawson in ref. 120:

‘…the key issue is more the precise control of the enhancement component, that we do understand well, namely the electromagnetic enhancement. That, in turn, requires very precise control of substrate fabrication that is highly repeatable and very affordable, a combination that has proved remarkably elusive over the years’.^120^

The above *Faraday Discussion* extract from ref. 120 also alludes to the debate over the exact enhancement mechanism in SERS *i.e.* how much of it is from ‘chemical effects’ – essentially alterations to molecular species being analysed as they adsorb to metallic surfaces. This is opposed to electromagnetic routes of enhancement, which are plasmon-mediated. While electromagnetic enhancement is undoubtedly by now understood as the predominant enhancement mechanism,^115^ chemical effects do exist. And while much more modest in comparison, the misunderstanding, or uncertainty, surrounding chemical effects can be off-putting towards the use of SERS in analytical space. To this end, we echo some of the commentary in ref. 120 that suggests ‘tying down’ the contribution for any chemical contributions to the enhancement for specific molecules. This may mean that SERS applications must be considered carefully with the specific analyte molecules to be detected in mind.

### 6.1. SERS applications

Despite the issues noted above, incorporation of SERS substrates into devices is not ordinarily prohibitive, SERS chips tend to be on the 10 mm^2^-scale, which could easily accommodate 10^7^ nanostructures depending on structure sizes/pitch and so forth. Many SERS substrates are often external to the Raman system, and as suggested above, this is likely to remain the case. However, even with an on-chip/waveguide device, SERS-enhancing parts can be easily integrated into the channel to boost the Raman signal. Despite the detections in the very low or even single molecule regime garnering interest,^121^ as Bell has recently suggested, the next step for SERS may be to prove its worth for routine analytical analyses.^122^ While SERS has been mooted as needing a killer app, potential applications have nevertheless arisen in medical diagnostics space. And as seen with the COVID19 pandemic, easy-to-fabricate lateral flow test strips, but with a SERS aspect, could make inroads.^123,124^

An emerging area in diagnostics are organ-on-chip (OoC) models, which seek to emulate various bodily organs on the nanoscale. OoCs conveniently offer an alternative to otherwise misrepresentative animal environments, and consequently failed drug developments, which do not always emulate human physiology. Moreover, OoC use sidesteps any ethical considerations. Similarly, *in vitro* analyses *via* cell cultures are often inadequate, where pathophysiological responses cannot be faithfully repeated due to, for instance, inaccurate inter-cell interaction or external interferences. Initially, starting as a lung-on-chip model, analogues to the blood–brain barrier, heart, gut, and many others have since appeared. However, integration with high-end, bulky Raman systems is challenging, and hence, SERS media incorporated into such systems offer viable solutions to increase sensitivity in OoC models.^83,125^ Further, the microfluidic nature of such systems could resolve the repeatability problems with many SERS analyses,^83^ averaging the SERS signal.

An emerging area in SERS sensing are wearable SERS media, where human-worn patches can be used to monitor biofluids.^126,127^ For instance, Yang Li *et al.* designed and characterised a wearable SERS patch with microfluidic channel and wicking paper for sweat analyses in athletes.^128^ The sensor incorporated a carbon tape layer to block incident laser light from skin interaction and reached a maximum temperature of 44.5 °C (from 37 °C) temperature increase, tolerable for *in situ* analysis. Further, the device suffered no delamination with significant strain and remained comfortable for human volunteers during use, with puncture testing showing a strength of 20 N for a 100 μm film, about twice as strong as those commonly used in food packaging. Koh *et al.* developed a portable technology comprising an outer sweat-absorbing layer, a SERS active layer containing a plasmonic silver nanowire, and a dermal protecting layer.^129^ The feasibility of the technology was demonstrated using 2-flurormethamphetamine (2-FMA) for drug detection. Similarly, Chung *et al.* fabricated a wearable flexible sweat pH-based sensor using a combination of electrospinning of thermoplastic polyurethane and gold nanofibers.^130^ The developed device required only 1 μL of sweat for measurements and indicated good repeatability. Mogera *et al.* also developed a label-free paper patch for continuous sweat rate and metabolite detection, including uric acid.^131^ Such technologies pave the way for various adaptations in wearable SERS for illegal drug surveillance and non-invasive drugs monitoring in patients, as well as the measurement of a wide range of health biomarkers beyond those found in sweat.

SERS has also found application in miniaturised immunoassays at the point-of-care in the detection of a range of diseases, such as those relating to the respiratory system. Liu *et al.* conducted a study to identify the presence of COVID-19 using a lateral flow immunoassay comprising functionalised two-layer silver-coated SiO_2_ nanoparticles as SERS nanotags.^132^ When coupled with SARS-COV-2 virus protein, diagnosis of IgM and IgG antibodies was achieved. Leong *et al.* has also detected COVID-19 using SERS but in the context for a miniaturised hand-held breathalyser for the mass screening of COVID-19.^133^ While work on integrating SERS into commercial products, or as an option for analysis as an accessory, many of these systems form a wider trend of using Raman spectroscopy in healthcare settings, at the point-of-care, where the technique is now showing tremendous promise in portable diagnostics space.

## 7. Point-of-care Raman devices

**Fig. 7:**
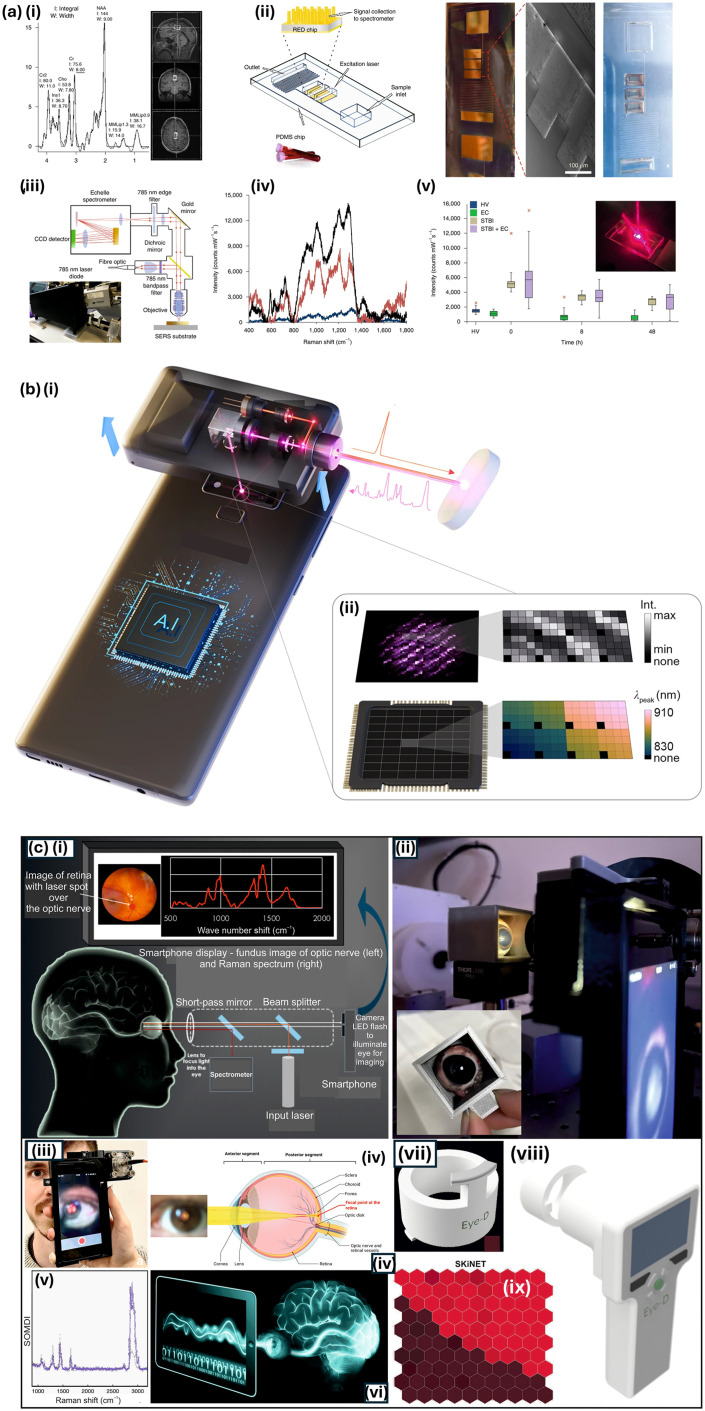
Point-of-care Raman systems. (a) Point-of-care micro-engineered device for traumatic brain injury (TBI) bio-diagnostics. (i) Neuro-medical imaging with magnetic resonance imaging (MRI), (ii) schematic of optofluidic chip indicating how one drop of blood moves through the device with three panels (right) of optical images of the chip, (iii) schematic of multiplexed system indicating analyses of biomarkers, (iv) acquired SERS spectrum compared with the pre-established biomarker fingerprints and (v) indicates how the biomarker level can be monitored over time using the developed optofluidic technology. Adapted/reproduced from ref. 148 with permission from Springer Nature, Rickard *et al.*, *Nat. Biomed Eng.*, 2020, **4**, 610–623, copyright 2020. (b) Smartphone Raman spectrometer and data processing for analysis of, for example, drug classification. (i) Smartphone spectrometer with identified band pass filter arrays attached to a rear camera with an attachable Raman system equipped with 785 nm laser. (ii) Images captured at chosen wavelengths encoded as a spectral barcode. Adapted/reproduced from ref. 144 with permission from Springer Nature under Creative Commons CC-BY-4.0, U. J. Kim *et al.*, *Nat. Commun.*, 2023, **14**, 5262, copyright 2023. (c) EyeD technology for rapid traumatic brain injury point-of-care diagnostics indicating concept (i) through to (ii) design of EyeD with 3D printed model, and (iii) lab prototype. (iv) shows the eye anatomy and (v) an acquired spectrum from a self-organising map (SOM) analysis, providing a ‘window into the mind’ (vi). And (vii and viii) engineered EyeD designs and (ix) visualisation of SOM map data separation into nodes of similar spectra. Adapted/reproduced from ref. 164 with permission from American Association for the Advancement of Science under Creative Commons CC-BY-4.0, Banbury *et al.*, *Sci. Adv.*, 2023, **9**, eadg5431, copyright 2023.

Point-of-care (PoC) Raman devices are small systems that are used to garner diagnostic information while in the presence of the patient and represent a significant advancement in the field of medical diagnostics, offering the potential for rapid, non-invasive, and highly specific identification of biological molecules, predominantly at the bedside or in outpatient settings (Fig. 7). PoC is thus especially valuable in environments where timely diagnosis is critical.^134–136^ The integration of Raman spectroscopy into portable, user-friendly devices has been driven by the need for on-the-spot diagnostics in diverse healthcare settings, including remote clinics, emergency rooms, and developing regions. The need is compounded by increasingly under pressure healthcare systems, whether because of the COVID-19 pandemic, aging population demographics, or lack of Government spending. Here, details of a PoC Raman system needed can be disease specific, however 750–1200 nm is of interest in biosensing as it avoids water absorption at the higher end (>1200 nm) and protein absorption below 750 nm.^94^

The primary aim of PoC Raman devices is to overcome the cost and mobility limitations of traditional benchtop spectrometers and produce portable, easy to use, and affordable devices which maintain performance. Recent advancements in optics, electronics, and miniaturisation technologies have ensured the capabilities of Raman have found applications outside the traditional laboratory, thus enabling rapid and on-site analysis across diverse applications.^136–138^ These typically compact and lightweight devices are equipped with user-friendly interfaces, making them accessible to specialist and non-specialist users alike. PoC devices, unlike traditional systems often integrate advanced features such as wireless connectivity, battery-powered operation and robust data analysis software, frequently utilising cloud-based services, that allow for real-time diagnostics and decision making in the field.^139–141^

With the transition from bulky laboratory instrumentation to highly compact systems, modern PoC Raman devices have advanced to include handheld Raman spectrometers such as Thermo Fisher TruScan™ RM, Agilent Vaya™, and B&W Tek TacticID™. Widely used in real-world applications including, hazardous and illicit compound identification, these ergonomically designed devices provide highly efficient tools for investigators. TacticID™, for example, has the ability to measure through packaging to avoid direct contact with the sample thus offering the flexibility to operate anywhere without compromising safety.^141^ Similarly, TruScan™ has been employed in pharmaceutical supply chains to rapidly identify counterfeit drugs.^140^ With colour-coded easy-to-read results, such technologies can now be used by non-technical operators to rapidly identify chemical compounds therefore eliminating the need for compendial ID testing, which requires specialised laboratory training and significant time.

Furthermore, smartphone-based Raman devices, leveraging the advanced camera technology in modern smartphones, can be used for PoC applications. For example, Rentzepis *et al.* have developed a smartphone-based Raman spectrometer system to analyse drugs and biological molecules.^142^ The setup incorporated a diode laser and a diffraction grating to record the Raman spectrum. With the smartphone placed behind and facing the transmission grating, the laser then illuminates the sample of interest with the camera subsequently recording the spectrum. Lebanov *et al.* applied a similar approach combining smartphone-based technology with machine learning for essential oil quality evaluation^143^ and Kim *et al.* identified unknown drugs with 99% accuracy using their smartphone Raman spectrometer to define a spectral barcode of each compound.^144^ While smartphone cameras have proved useful, their performance is typically unsuitable for routine Raman analysis, which is a weak effect (few photons). In addition, a poor dynamic range, high noise, and possible differences in performances between handset batches could be problematic – issues representative of detectors optimised for photography rather than scientific measurement. Should some of these criteria be relaxed, however, *e.g.* a qualitative identification or semi-quantitative measurement specified rather than quantitative one, smartphone-based systems could be useful as an element for widely accessible Raman analysis.

PoC Raman devices present multiple advantages over traditional Raman instrumentation. Firstly, in their portability, with devices like TruScan™ and Agilent Vaya™ weighing 0.9 kg and 1.62 kg respectively, and combined with their user-friendly designs, are well suited for both bedside and field use.^140,145^ Perhaps the single most advantageous use of PoC Raman devices is in their ability to operate in regions without the need for extensive laboratory infrastructure, enabling access to diagnostics in remote geographical and underserved or low-resource locations. The non-invasive and label-free ability of Raman spectroscopy lends itself well to PoC devices, reducing the overall costs and sample preparation time. For example, Raman systems have been used to directly analyse saliva or blood plasma, bypassing complex biochemical assays. Fujihara *et al.* successfully discriminated human and non-human blood using a PoC Raman device directly measuring blood spots up to three months old, which is of particular use at a crime scene where unknown blood stains are identified, and subsequent analysis must be performed on location.^146^ Moreover, with rapid data acquisition (often under 10 seconds) PoC Raman devices allow medical professionals to make on-the-spot decisions in critical care settings, such as identifying *N*-acetylaspartate, a traumatic brain injury (TBI) biomarker or cardiac troponin I to evaluate cardiovascular health.^147,148^ Raman spectroscopy provides molecular-level identification of analytes, of particular advantage in biomarker detection or discriminating illicit drugs.

Elsewhere, miniature Raman devices have also been developed for the detection of different skin diseases, including cancers and atopic dermatitis. Lightnovo ApS fabricated a mini-Raman™ device capable of providing molecular composition of the skin up to several hundred micrometres in depth. With dimensions similar to those of car keys and limit of detection (LoD) values of 0.1 g L^−1^ for *p*-coumaric acid, the technology is well-suited to clinical practice and for monitoring of disease.^149^ Similarly, Horiba Ltd. have designed and fabricated two mini-Raman spectrometers; the CC-Raman-NIR spectrometer and the Mini-CCT+ for various applications within life science, forensic and security, and pharmaceutical and cosmetics.^150^

Perhaps one of the most significant advancements in PoC Raman technology is the incorporation of SERS, which uses metal nanostructures to enhance signal sensitivity^151^ (also see section 6). SERS has expanded the applicability of PoC Raman devices by enabling the detection of trace-level analytes, such as biomarkers, contaminants, and drugs, in complex matrices such as blood, saliva and urine.^136,137,146,152–156^ Portable microfluidic Raman systems are often integrated with SERS chips, designed for single-cell analysis and real-time diagnostics. Rickard *et al.* developed a portable optofluidic chip using SERS to detect *N*-acetylaspartate in patients with traumatic brain injuries (TBIs).^148^ The authors use electrohydrodynamic lithography, a technique exploiting instabilities in polymers caused by an applied electric field gradient, to fabricate sub-micrometre gold-covered pillars in an optofluidic chip to identify picomolar concentrations of TBI biomarkers. Elsewhere, Wang *et al.* developed a portable and ultrasensitive, recyclable SERS-microfluidic biosensor for the detection of uranyl (UO_2_^2+^) ions, achieving detection limits as low as 7.2 × 10^−13^ M.^157^

Despite the clear advantages and benefits of PoC Raman devices, they are associated with several limitations and challenges preventing their widespread use, particularly in the healthcare setting. Firstly, Raman scattering is inherently weak, making it challenging to detect low-abundance analytes. In biological samples background fluorescence can further obscure Raman signals, for example in blood-based diagnostics autofluorescence often overwhelms the signal thus extensive signal processing or SERS substrates are necessary to enhance detection. This brings about a further limitation in terms of overall cost. SERS substrates, requiring nanoscale materials can be costly to produce and are often single use, therefore significantly driving costs for routine clinical testing. Moreover, the listed capabilities of Raman including the non-invasiveness, label-free, and non-destructive nature of the technique, come at the cost of extensive requirements for the instrumentation, *i.e.* the laser must have a stable wavelength and optical power, there should be low noise associated with the spectroscopic sensor and, the optics within the spectrometer should have a large clear aperture. Rentzepis *et al.* have applied right-angle geometry in their device to try and circumvent the noise and interference from Rayleigh scattered light – photons without an energy/wavelength change.^158^

The future of PoC Raman devices lies in their continued miniaturisation, cost reduction, and integration with digital healthcare platforms. Developing robust, low-cost SERS substrates and eliminating the need for sample preparation will be pivotal for widespread adoption.^159,160^ Moreover, regulatory approval for clinical applications remains a slow and expensive process with devices subject to stringent criteria for sensitivity, specificity, accuracy and reproducibility with many technically ready PoC devices failing to reach markets due to unresolved regulatory hurdles and therefore, such frameworks need to continue to evolve to support the commercialisation of PoC devices. While challenges remain, ongoing innovations in nanotechnology, advances in data analysis strategies^161–164^ including artificial intelligence,^165,166^ and system engineering (see section 8) promise to address limitations.

### 7.1. Microfluidic Raman devices

PoC devices often incorporate microfluidics, which refers to the manipulation of fluids on the micron-scale leading to small, highly integrated sensors often performing multiple functions.^83^ Raman microfluidic chip architectures can vary greatly, notably incorporating many different strategies for analyte molecule selection and/or concentration including optical trapping, electrical effects, and mechanical features.^167^ Recently, Persichetti *et al.* developed a multifunctional optofluidic lab-on-a-chip platform for Raman analysis, integrating a micro-jet waveguide in their efforts to miniaturise the technology.^168^

The promise of SERS and microfluidics has recently been surveyed in a *Lab on a Chip* review article,^83^ where the authors point out distinct advantages in terms of low reagent volumes, faster diffusion rates, and easier manipulation of reagent with pressure, electrical and magnetic effects amongst others.^89^ Microfluidic devices have generally required properties, such as sealed interconnects and suitable thermal properties as well as the chemical stability of materials.^169,170^ Whereas microfluidic platforms have traditionally been made using glass and conventional lithography, other options are emerging, namely stereolithography 3D printing, which uses UV or green wavelength light to harden polymer resists from specified computer aided design (CAD) models, to make fluid channels with diameters on the tens μm to mm-scale.^171^ We note that 3D printing brings with it its own problems, such as rough channel wall textures and lack of reproducibility. The material must be optically transparent at the laser wavelength(s) required and have low fluorescence background – key in the low volume and low analyte concentration regime. Comment on emerging polymer material choice, for examples PMMA or PDMS, is given in ref. 83 with notes on flexibility and electroosmotic flow where an applied electric field attracts ions, which then also move the solvent molecules. SERS and microfluidics continues to be a hot area within bio-studies with different research groups recently reporting *E. coli* detection *via* aptamer-mediated silver nanoparticle synthesis *in situ* (*i.e.* AgNO_3_ reduced by a suitable reagent within the microfluidic channel)^172^ and secondly, *via* a piezoelectric transducer generated acousto-fluidic set-up.^173^ In the latter case, the authors note the possibility of bacterial multiplexing *via* multiple SERS tags for different bacterial species.

One clear advantage in a SERS context is the averaging of signals through the solution flow, mitigating the large variations associated with SERS measurements, and making SERS a more attractive analytical option,^174^ offering proper quantification of a target molecule. Going forward, key challenges remain on re-usability of microfluidic SERS platforms and combination with other analytical techniques.^83^

Similarly, the nature of microfluidic flow means that higher power densities can be used without concern over causing sample damage where heat energy is quickly dissipated and analyte molecules rapidly replenished in the focal region. For this reason, (high-power) near-infrared laser use *i.e.* 785 nm (typically) with a small focal volume *i.e.* >40× magnification, say, can be used without reservation.^167^ Nevertheless, a prominent issue with microfluidic channels remains in the adherence of analyte to channel walls, which can then contribute to the observed Raman signal, undermining quantitative determination. This is a common problem appearing in low concentration SERS studies in general, and warrants, for example, careful consideration of how low-concentration solutions are made in sequential solutions (dilutions) in an analytical investigation (analyte molecules sticking to the glass vial walls). In a microfluidics context, this problem can be avoided with two-fluid flow where an oil layer is incorporated.^175,176^ Alternatively, disposable, ‘few-use’ microfluidic chips can reduce concern over chip reuse and memory/fouling effects.^167^ Such systems, then, will have laser excitation and Raman detection off-chip, which is an easier arrangement but may be less convenient than a fully integrated Raman chip.

### 7.2. Point-of-care Raman devices summary

In summary, Raman systems have emerged as powerful tools in the field of biomedicine, offering non-invasive, portable, label-free, and highly sensitive analysis of biological samples. These compact systems leverage the principles of Raman spectroscopy, and such miniaturisation has revolutionised their applicability, allowing for portable, real-time analysis in, for example, clinical settings, point-of-care diagnostics, and *in vivo* monitoring.^177,178^ The integration of miniaturised Raman systems in bio-applications has opened new avenues for early disease detection, pathogen identification, and the analysis of complex biological fluids to name a few. Their small size, coupled with advances in photonics and data processing, enables the deployment of these systems in various challenging environments, where the complexity and/or size of traditional systems is obstructive, from bedside diagnostics to field-based studies. Many of these miniaturised PoC chips incorporate microfluidics, perhaps in a SERS context. As a replenishable source of analyte molecules, this then perhaps offers a realistic path to commercialisable technology (as opposed to fully integrated Raman chips). Further, we note the impact that 3D printing can play in the fabrication of these microfluidic devices, with a trend for smaller *i.e.* μm-sized parts. However, smaller is not always useful – channels on the smallest of scales may need to have their fluid dynamics, and hence analyte–solution mixing carefully (re-)considered. Alongside this, micro-dimension printing can be slower and is more expensive, perhaps limiting scalability for manufacture.

Portable medical devices represent a hot area, and optical-based systems can form a key part of remote, out-of-the-clinic diagnostics. However, what about the role of Raman miniaturisation, specifically? Regulatory barriers will form an expected pushback, as is the case for any medical implementations, but the signs are positive. A recent report, for instance, details the use of multi-wavelength Raman as useful for Alzheimer's diagnosis at >93% accuracy. The UK-based group now intend on making a suitable portable Raman device.^179^ Measurements directly on patients might pose more of regulatory problem; analysing external fluids are more straightforward from a regulation standpoint, although problems can still arise in biofluidic storage, pretreatment, and with inherent variations in composition within classes *i.e.* intra-class variance.^14^ Should such issues be resolved, Raman spectroscopy could target an array of health conditions.^155^ While the AI revolution will undoubtedly accelerate progress in many fields of science, it might do particularly well in clinical spectroscopy, where especially information-rich datasets are often generated but that can be difficult to decipher given their complexity. We note the particular complexity of bio-samples, which can contain an array of molecular species alongside the biomarkers of interest. The inherently sharp Raman peaks permit optimal multiplexed detection and arguably thus can couple well with AI and machine learning techniques that can pick out, perhaps hitherto unappreciated, spectral features with diagnostic significance. Aside from advanced data analysis strategies, there is a push for not just portables but wearable devices perhaps additively manufactured, and this could drive Raman instruments in a PoC context right down in size, including wearable SERS chips,^127,180,181^ perhaps. However, to do so successfully in a competitive application space, such as PoC device development, Raman systems should be considered holistically from the outset – systems engineering – to avoid roadblocks to clinical adoption downstream.

## 8. Systems engineering

While the reduction in size of Raman systems confers many benefits, opening up opportunities across a broad range of applications, there are a number of broader aspects to consider when designing a compact, efficient, and reliable Raman system, including its performance, size, weight, power consumption and cost (SWaP-C). In terms of software and data, the availability of spectral databases, and algorithms for rapid data processing, is also critical. Spectrometers are an example of optical system, and the field of systems engineering ensures the optimal functioning of such systems by considering not just the optics but, the performance requirements, durability, usability, the SWaP-C criteria, alongside other concerns in hardware, software, integration, testing, reliability and operation.

Systems engineering can be defined as a transdisciplinary and integrative approach to enable the successful realisation, use, and retirement of engineered systems.^182^ This relates to combining multiple interacting elements in an organised manner to achieve one or more purposes. In the case of Raman spectroscopy, it covers combining optical, electrical, mechanical, and software subsystems into an optimal device. The relationship between the subsystems should be considered from the outset, and interfaces identified and documented. While Raman laser wavelength used and detector are normally the first to be considered in the optical aspects of miniature Raman devices, the chosen components can have an acute effect on other system aspects. For instance, a longer (redder) wavelength *e.g.* near-IR, 785 nm, laser will require a detector with a larger spectral range, as the Raman peaks will be more greatly shifted from the Rayleigh laser line (and more spectrally separated). This can also affect device size (dispersion element to detector distance must be considered). Adjusting the optics can be useful here, but subsequently in turn affects light throughput *i.e.* signal intensity, and resolution – a higher numerical aperture lens will mean more obliquely incident photons collected. And so forth, the system thus must be considered holistically, and optimised with interdependencies in mind.

Moreover, a life cycle model may be employed to illustrate the sequence of different stages of the process – from initial conception to retirement, such as the ‘V-model’. In this context, the initial optical systems engineering process includes: requirement analysis, feasibility studies (realistic technical/cost possibility), system architecture development, trade studies (comparing the pros and cons, trade-offs for different device designs), definition of subsystems and interfaces, detail design and analysis, component specification, tolerancing (what are the permissible limits in the system where the performance remains acceptable? *e.g.* mirror tilt ±0.1°?, laser wavelength ±1 nm?) and error budget allocation (for any one tolerance, how much each contributing error is allowed *e.g.* for laser wavelength, contributions from thermal drift, calibration error, grating fabrication differences *etc.*), vendor monitoring (checks on one's suppliers), assessment of facilities/equipment, fabrication and inspection of parts, component testing, assembly, alignment and verification. Detailed discussion on the process is available in ref. 183. The process is essentially deciding what is needed and whether it's feasible to meet such needs in a device, followed by a comparison of options and detailed design and testing. One observation we would make is that the barriers to commercialisation often come from unexpected and unforeseen sources, for instance, while focus is often on device performance and the bill of materials required, the engineering may prove problematic, where alignment procedures, say between optical interconnects on-chip, can prove to be prohibitively difficult and thus expensive.

Specific considerations for Raman spectroscopy include optical throughput, spectral range and resolution, requiring compromises when developing a miniaturised system, with different approaches to the size reduction – from optical bench to compact product, or building up from miniaturised components.^77^ Example subsystems and considerations in this context are outlined in Table 1. While many of the optical aspects of systems engineering in Raman system design are arguably well-appreciated by those in Raman spectroscopy research, as exemplified in a recent review article on laser wavelength use with Raman,^21^ other aspects pertaining to the electrical, mechanical, and software aspects (see Table 1) may not be.

**Table 1:** Overview of the subsystems and considerations and requirements

Sub system	Optical	Electrical	Mechanical	Software
**Considerations and requirements**	**Laser selection**: excitation source – power, wavelength, stability, safety	**Power consumption**: optimise illumination, detection, embedded processing	**Compact dimensions**: balance performance and size	**Rapid data processing and analysis**: including software to quickly process spectra and match against databases, algorithms for baseline correction
	**Optical path optimisation**: use of fibre-optics, compact optical elements	**Embedded computing**: potential to embed a compact, efficient processor for real-time data acquisition and analysis	**Ruggedisation**: withstand relevant environmental conditions – temperature fluctuations, humidity, dust, and physical shocks	**AI integration**: potential to incorporate AI-based spectral processing –enhance accuracy, automate classification
	**Detector selection**: high sensitivity, low noise, fast response (CMOS, CCD)	**Connectivity**: low-power, robust connectivity for *e.g.* cloud-based databases, internet of things integration, real-time updates	**Usability**: lightweight, easy-to-use controls (support non-expert operators, use by personnel with protective gear)	**User interface**: intuitive, functional, streamlined interface for operation
	**Optical filtering**: filtering to isolate weak Raman signals (*e.g.* notch, edge)		**Thermal management**: appropriate heat dissipation for lasers and detectors – stable operation in various environmental conditions	
	**Spectral resolution**: high resolution Raman peaks – grating selection, optical alignment			
	**Background minimisation**: mitigate fluorescence *via e.g.* time gating, excitation wavelength selection			
	**SERS substrate selection**: maximise SERS signal – enhancement, reproducibility, cost			

Together with outlining and designing the subsystems and interfaces, it is also critical to develop test plans with a view towards verifying that the outlined system requirements are met. Moreover, it is critical to take the perspective of the end user when designing a Raman system, to ensure the developed hardware and software meets the specified requirements, which flow down to the subsystem and component performance. One pertinent example might be analytical sensitivity *versus* dynamic range, for instance. Here, a user might conceivably be keen on a device operating a wide range of concentrations rather than one achieving the lowest detection limit possible. Similarly, proper quantification is not always needed, instead semi-analytical analyses may be sufficient, supported by a confirmatory second-stage analysis in a lab setting. This might be a good pointer for SERS which, unlike conventional Raman spectroscopy, draws debate over its nature as a properly quantitative technique. Furthermore, Table 1 encompasses a range of general considerations Moreover, a photonic integrated circuit-based Raman system entails many specific details in terms of photonic chip design, testing and verification and relying on a supply chain incorporating design, manufacturing, packaging, testing and system integration.

Evidently, designing an efficient, miniaturised Raman spectrometer requires careful systems engineering. Issues of consistency in final product, which while often a side thought within research, are critical when it comes to the final device equipment. Similarly, while durability and robustness/ruggedness fall distinctly into the later stages of development for products, they are essential for a portable system. Therefore, design of systems is often vastly different enterprise from that of scientific research where many other aspects come into play pertaining to late technology readiness level (TRL), product finalisation stage and end-user requirements, rather than fundamental and exploratory investigation.

### 8.1. Digital systems design

The big shift in systems design is to ‘the digital’ where computer models can be used for digital design facilitating rapid iteration and more adaptive design. In its most developed sense, this comes in the form of digital twins, highly accurate computational representations of a real-world system that can change in real time in response to outside/real-world events. Similarly, modifications to the computer model can often influence the actual system, giving a genuine two-way interaction. This model-based systems engineering (MBSE) then facilitates integration across the various disciplines (*e.g.* electrical, mechanical design), promotes change management and traceability, with the formation of a ‘digital thread’. The next challenge may be in more open-source tools that can be easily integrated into and across different digital workflows.^9^

Recent advances in digital technologies, particularly in embedded processing, edge computing and cloud-based analytics, are increasingly shaping how miniaturised Raman systems are designed and deployed. However, the growing reliance on software should not be viewed as a substitute for careful optical and systems engineering. While machine learning can compensate for variability to a degree, it cannot recover information that was never captured due to insufficient signal-to-noise, poor spectral fidelity or unstable excitation. In this sense, digital tools are most effective when used to amplify well-designed hardware, rather than to rescue marginal designs. A key systems-level challenge therefore lies in defining clear boundaries between what must be guaranteed by physics and engineering and what can legitimately be delegated to algorithms. As Raman devices move further into non-expert and regulated settings, this distinction will become increasingly important for validation, trust and long-term adoption.

## 9. Key challenges, market insights and future directions

### 9.1. MEMS, integration and fabrication challenges: maintaining performance

A large part of the ongoing challenge is making and packaging small components for Raman systems in a way amenable to practical, commercially viable systems. And so, while MEMS technology has significantly contributed to the advancement and miniaturisation of Raman spectroscopy, several challenges remain. One of the challenges in MEMS-based Raman systems is achieving high signal sensitivity; today's handheld/portable Raman instruments typically report signal-to-noise ratios (SNR) of ∼300–700 : 1 on standard samples (*e.g.*, polystyrene at 1001 cm^−1^), which is adequate for identification but can be limiting for trace quantification in complex matrices. On integrated photonic platforms, waveguide-based Raman spectroscopy has achieved limits of detection down to 0.03 mol L^−1^ (≈0.2 wt%) for isopropyl alcohol in water without any surface-enhancing substrate, illustrating the progress but also the gap to trace-level sensing.

Fluorescence suppression remains pivotal: SERDS can deliver ∼15× improvement in signal-to-background-noise for in-field samples, and recent optimisation studies define practical dual-line wavelength separations (≈2–4 nm at 785–830 nm) for robust reconstruction in highly fluorescent biological tissues. Against these benchmarks, a desired sensitivity for miniaturised, MEMS-enabled Raman can reach <0.01 wt% (100 ppm) in liquids and ppm to sub-ppm in gases for many environmental/biomedical applications; the latter is already routine in integrated photonics using on-chip mid-IR absorption (*e.g.*, 0.3 ppm methane with slot-waveguides), setting an aspirational target for Raman platforms that waveguide Raman seeks to approach through low-loss waveguides, long interaction lengths, and sorbent claddings. Reducing noise levels and improving detection limits require innovations in optical coatings (high-extinction stop-band filters), low-Raman-background materials such as SiN/Ta_2_O_5_ and MEMS fabrication techniques (sidewall-roughness control to minimise scattering loss). Additionally, MEMS devices need to endure harsh environments and mechanical stresses, thereby necessitating the use of advanced materials; for example, boron-doped polycrystalline diamond micro-hotplates can operate up to ∼2731 K at ≤100 mW, greatly improving durability/thermal stability for Raman probes and temperature-programmed measurements – but at present they are expensive to manufacture and their extreme-temperature operation must be managed to avoid cumulative graphitisation.

Furthermore, the integration of MEMS devices into complete Raman spectrometers demands precise on-chip alignment/calibration to maintain optical performance; wafer-level sealing, fibre-array attachment, and flip-chip bonding from silicon photonic MEMS platforms are emerging to improve reliability and scalability, while ‘swept-source’ Raman using MEMS-tunable lasers (VCSELs) has demonstrated up to ∼1000× optical throughput gain *versus* dispersive portable spectrometers at eye-safe powers (∼1.5 mW), shifting complexity from bulky spectrometers to chip-scale light sources and enabling uncooled detectors. Recent advancements also include hybrid MEMS filters capable of operating across multiple wavelength ranges, enhancing the adaptability of Raman devices for diverse applications.^54,55^ Integration with silicon photonic platforms is emerging as a key trend, where MEMS Fabry–Perot interferometers are combined with waveguides and couplers for fully on-chip Raman spectrometers, reducing alignment complexity and improving robustness. Actuation mechanisms such as electrostatic and piezoelectric drives now enable sub-millisecond tuning speeds, supporting real-time adaptive spectroscopy. High-reflectivity dielectric coatings (*e.g.*, SiO_2_/Ta_2_O_5_ stacks) are being optimised for thermal stability and minimal scattering loss, critical for harsh environments and field deployment.

The availability of low-cost, reproducible components for small Raman devices is being facilitated by developments in lithography, including new techniques that can maintain fidelity at high throughput;^8^ MEMS are still often fabricated by conventional photolithography in the micron-scale regime^184–188^ and pushed down to tens of nanometres using X-ray/e-beam patterning,^189–192^ while nanoimprint lithography (NIL) – which uses an electron beam-made master and an inverse sub-master to emboss nanostructures – has matured industrially for high-throughput, low-cost patterning^193^ despite early lab-scale imperfections.^194^ Patterning using additive manufacturing (3D printing) techniques like two-photon polymerisation and stereolithography is not fully industrialised yet, but they show potential for future use with their accuracy and reliability.

However, moving parts as in MEMS devices can be a problem for scalability, which must be considered early-on in any device development endeavour; nevertheless, MEMS-based systems can light the way for more integrated systems, with future scope including full on-chip Raman: monolithic MEMS-tunable lasers, on-chip filters/spectrometers (*e.g.* multi-aperture SiN FTS with 0.5 nm resolution on 200 mm wafers), integrated detectors, automated alignment, and embedded techniques such as SERDS and SSE (thermal shifted-excitation using micro-heaters) and AI-assisted denoising, all aimed at reaching ppm-level quantitative sensitivity in compact, robust, and manufacturable devices. Smaller spectrometers and MEMS developments within Raman research feed into the ultimate goal of a fully on-chip Raman system, and while challenges persist (fluorescence, filter extinction on chip, detector dark noise, vibration robustness, packaging), the trajectory of integrated photonics and MEMS strongly suggests practical, field-deployable instruments with trace-level sensitivity are within reach.

### 9.2. SERS outlook: analytical SERS and SERS-based devices

Elsewhere, specific fabrication challenges remain in SERS nano-architectures, where nanometric variations to structural designs can dramatically affect the measurement sensitivity and reproducibility. The upshot of this then hangs the question of SERS' utility as a properly quantitative technique – a continuing research area that sparks fervent discussion. While the use of SERS is undoubted for screening purposes or ‘first-indication analysis’ otherwise, where a qualitative identification can suffice, calibration techniques may be needed for quantitative SERS.

With respect to system integration, SERS substrates are more likely to persist as modular, replaceable components rather than being permanently embedded within Raman devices. From an engineering and translational perspective, this separation preserves flexibility, reduces system complexity and avoids imposing the material and shelf-life constraints of plasmonic nanostructures on the core optical platform. However, limited forms of integration may still emerge in tightly controlled contexts, for example where SERS structures are confined to sacrificial microfluidic cartridges or disposable sensing heads. In such cases, the Raman system itself remains agnostic to whether enhancement is present, while the SERS element is treated as a consumable assay rather than a permanent optical component. This framing aligns more naturally with existing analytical workflows and cost models and avoids the expectation that SERS must function as a universally quantitative technique across all applications.

### 9.3. Analytical options and standardisation

It is worth noting there are a multitude of alternative techniques to Raman. Infrared (IR) absorbance is another vibrational spectroscopy which has, historically, better established technological norms. Regardless, IR offers complementary information, sensitive to changes in molecular dipole moment as opposed to the polarisability change in Raman scattering. Different spectral peaks are thus present. Besides, portable mass spectrometries are also available *e.g.* gas chromatography mass spectrometry (GC-MS). Direct analysis in real time mass spectrometry (DART-MS) and rapid evaporative ionisation mass spectrometry (REIMS) systems have found use in food security applications.^195–197^ Fully elemental options also exist such as X-ray fluorescence (XRF) as well as laser-induced breakdown spectroscopy (LIBS), which uses laser light to ablate a small region on a sample of interest. Portable nuclear magnetic resonance (NMR) systems have also appeared. These kinds of carriable systems are ruggedised for outside of the lab use and tend to have integrated data analysis platforms offering easy actionable results.^43,198^ Surface plasmon resonance (SPR) is a well-established, fast, and highly sensitive technique,^199^ which can be more cost-effective than other analytical techniques.^28^ The approach, like SERS, also relies on resonant collective electron oscillations. However, in SPR, a change in the spectral resonance position of these surface-confined, collective electron-light excitations, surface plasmon-polaritons, at a planar interface, or nanostructured surface, is brought by a change in the ambient refractive index from the addition of an analyte of interest. SPR requires surface modification (not necessarily the case in SERS) leading to unsuitability for detection of unknowns and possible problems working with complex bio-samples.

Thus, where Raman outshines other spectroscopies is in untargeted detections, where the analyst is working with a complex matrix, or worse, is completely oblivious to unknown substances in a complex environment. Here, Raman scattering spectra, with suitable interpretation, can provide an accurate assessment of the detected compounds.

Raman, categorised alongside mid-infrared spectroscopy, is labelled as mainly qualitative in its application to date, finding uses in pharmaceutical, explosives and narcotics detection. This is in contrast to near-infrared (NIR) absorption spectroscopy, which has found extensive quantitative use, for example in foodstuffs analysis. The lines, however, may be becoming increasingly blurred, and Raman and NIR can be seen as competitors. There is a significant push for Raman spectroscopy to be used as a properly quantitative analytical tool by industry, facilitated by groups, for example, such as the European Union-funded Characterisation and HARmonisation for Industrial Standardisation of Advanced MAterials (CHARISMA) project, seeking to ‘harmonise Raman Spectroscopy for characterisation across the life cycle of a material, from product design and manufacture to lifetime performance and end-of-life stage’.^200^ There is a particular need for metrological standardisation, particularly for miniaturised systems, with prior standardisation measures having been successfully applied to laboratory-based systems.^201^ A metrological infrastructure is key to ensure confidence in Raman measurement data, meaning outputs from Raman devices can be accurate and comparable.^202^ This includes not just wavenumber calibration for example with a certified reference material, but also detector calibration to correct for relative peak intensity differences between different kinds of detectors.^201^ Progress in Raman standardisation can align with a movement in standardisation in photonics packaging – the integration of optical components into robust, scalable devices assisted by advances in photonic foundries and spurred on by a wide range of applications for optoelectronic systems.^28^

### 9.4. Hybrid systems

Hybrid systems combine Raman spectroscopy with other analytical techniques to provide more comprehensive analysis capabilities. Recent advancements in hybrid systems include the combination of thermogravimetric and Raman systems for simultaneous analysis during the heating process^67^ and a MEMS-based spectrometer that integrates Raman and laser-induced breakdown spectroscopy (LIBS).^203^ The latter system uses MEMS mirrors for precise beam control and MEMS Fabry–Perot interferometers for dynamic wavelength selection, enabling simultaneous molecular and elemental analysis. This hybrid Raman-LIBS system can focus up to about 40 nm, has a lateral resolution of about 700 nm for Raman maps and 9.5 μm for LIBS maps. These systems can leverage MEMS mirrors, filters, and actuators to achieve high-resolution mapping of both molecular and elemental compositions.

Hybrid systems are particularly valuable in applications such as space exploration, industrial inspections, biomedical diagnostics, and food safety, where multi-modal analysis can provide more comprehensive insights.^204^ While arrangements combining SPR and Raman are possible, their implementation is often impractical^205^ and not enough analytical benefit has been observed to push development beyond academic research. Similarly, multi-wavelength portable Raman systems *i.e.* more than one laser, or combined IR-Raman systems have not appeared extensively in the market. In the latter case, for example, a cursory search reveals an IR-Raman combo system from BaySpec Inc. (CA, USA).^206^ Thus, while the combination of different analytical techniques with Raman is undeniably useful, for instance appearing in the agri-food sector,^207^ the implementation of dual- or multi-spectroscopic systems in one device is perhaps highly specialist in application, limited to niches and custom-built requests, and therefore seemingly unlikely to make widespread market impact for the moment.

### 9.5. Convergence and device translation

Beyond a discussion on multi-functional Raman systems is the point that the development of miniaturised Raman spectroscopy devices is itself nevertheless still a multi-disciplinary effort, requiring the collaboration across disciplines and skills sets from optics and photonics, to packaging and materials science. An on-chip Raman device might be an especially good example of this, incorporating a need for miniature optics, on a compound semiconductor base, packaged together, and possibly with some of the other additionals outlined in this review, like microfluidics or plasmonic materials. These systems generate swathes of data and thus may require a resident AI/data analysis team. Further, applied science is also crucial where the driver for these handheld or on-chip systems is an array of applications in healthcare, homeland security, and food safety and an understanding of these driving application-spaces might be key in order to produce devices that have the optimal performance specifications. And as outlined in section 8, an array of systems considerations from engineers must also be considered before delivering a final product.

Therefore, the development of such devices would appear to fit nicely within the scope of convergence centres which seek to combine different branches of science, across Government, industry and academic partners, aligning with solving a ‘real-world’ problem. While the term convergence might be used, perhaps fairly, in a rather loose fashion to describe any multi-disciplinary effort, it tends to be applied for ventures on the larger scales, less time-bounded projects (or at least with some continuity beyond the original project), and more likely operating at the mid to late TRL scales, for translational research activities, in a more integrated, mission-driven fusion.^9^ But regardless of the organisation and focus of a convergence centre, what is clear is that more complex interdependent engineering problems require increasingly interdisciplinary co-operations in order to drive devices to market.

This review does not delve into the precise workings of translating Raman research into products (to do so would require a book!) but we note, in brief, some of the complexities in setting up and managing academic–industry partnerships in shared science spaces, including the usual discussions on intellectual property (who owns the research when it's funded by industry? Can it be licenced back to the industry partner and on what terms?). The exact role of the university is a good question – should it be confined to more disruptive R&D or can it overlap with less speculative research activities (fine iterations/optimisations) at the later TRL stages? Can senior academics devote significant time to industry consultancy? And how might these kinds of activities fit in with academic metrics – can translational activities be acknowledged in some way as journal papers and grants are?^208^ Regardless of the answer to these questions, academic–industry involvements are not just useful but critical to successful device developments, including the multi-faceted domain of miniaturised Raman devices, and may carry with it additional benefits such as graduate students, with key technical knowledge, joining industry partners (or a university spin-out start-up company) post-degree to develop the Raman devices further. We note that the involvement of a spin-out is an especially good idea, rather than established external companies, where start-ups are more likely to prioritise disruptive products, and therefore arguably align more congruently with university R&D activities.^208^

The nature of the translation from lab-based research at the lower TRL scale to usable products often starts at an early point. Many of the pains of this transition can be smoothed by a sound systems design approach from the outset. Suitable technical oversight, and insight, from the start, is thus critical. While not all potential bumps to commercialisation are foreseeable for specific device developments, many of the problems with taking a Raman device to market can be very general – aspects such as the laser wavelength used and detection efficiency are likely foci in academic research, while later stage developments are more likely to be concerned with aspects such as exact device size, power, and heat management, mechanical alignment, robustness/ruggedness, cost, and regulatory requirements. Thus, there must be a balance between performance and commercial viability.^209^

### 9.6. On-chip Raman as a convergence technology within the UK photonics and materials ecosystem

**Fig. 8:**
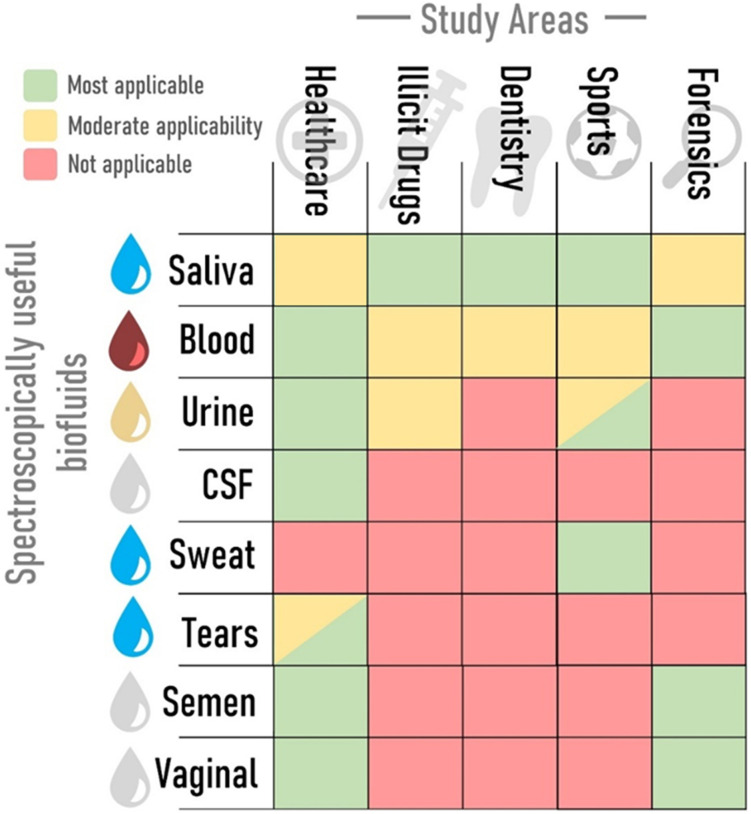
Estimation of utility of various biofluids for Raman liquid biopsy to different application areas. CSF = cerebral spinal fluid.

On-chip Raman genuinely offers a potential convergence of technologies in the form of (nano-)photonics, packaging, and compound materials (such as InP) and development of the smallest Raman spectrometer devices need careful consideration of these multiple fields. This is against a backdrop of increased emphasis on remote sensing needs, for example decentralised medicine, perhaps liquid biopsy in the form of biofluid screening for disease or wearable sweat sensors for athlete monitoring in sports. At present in the UK, there is a drive for more community healthcare hubs, for instance. Elite level sports, for example, Premier League football, have seen a massive uptake in player performance monitoring in the past 15–20 years, and this may continue, becoming increasingly sophisticated. Further application areas present themselves in dentistry, forensics, and drug detection (Fig. 8). Beyond biofluids, applications exist in national security and clandestine sensing in improvised explosives, or food security and portable detections in the wake of an increasingly global food chain. Alongside this there is a need for a new wave of ‘holistic’ scientists and engineers, who can work across some of these converging domains.

The authors note the recent advent of the Microelectronics UK event in London^210^ and the co-location of Photonics UK, and Embedded Systems UK, where the synergy of these domains might be key to the advancement of miniaturised photonic systems, including Raman devices. Increased collaboration between academia and industry can be viewed as essential to achieve these aims. This has been evidenced by the UK Government's recent investment on collaborative science in the form of the £42m (∼$56m USD) UK Research and Innovation –funded, Smart Nano NI project in Northern Ireland – one of the UK's constituent regions. The project, of which a number of the co-authors of this paper belong, operates broadly within photonics, nanotechnology and smart manufacturing, represents a paradigm shift in how research and development can work as it has sought to establish an ecosystem within which products can be developed across the technology readiness level (TRL) scale.^211^

Furthermore, the development of miniaturised spectroscopy is embedded within developments within larger trends within photonics, and materials science. As reported in the recent 2025 UK Photonics Leadership Group report, photonics is gaining significant traction as a field, underpinning some 13% of global innovation,^212^ of which spectroscopy constitutes a considerable share. In light of this, Innovate UK, a national innovation agency, has recently been holding a series with sector leaders in the form of a *Future Tech Deep Dive*, including a session focusing on the future of Photonics (October 2025, Belfast, UK) to garner future trajectories in emerging photonic technologies.^213^ The UK has also recently announced the set-up of a UK Semiconductor Centre as a way to amalgamate current efforts in semiconductor research and draw in external collaboration, for instance South Korea, which may offer manufacturing and scalability capabilities to complement the UK's expertise in research and development and packaging.^214^

### 9.7. The portable Raman device market

Predicting market trends and needs is far from simple, and some of the developments in miniaturised Raman may be prospective. *MATRIX*, the Northern Ireland Science Industry Panel, recently released a report on ‘Photonics and Lasers Capability’ (October 2025).^215^ Whether within a Northern Irish, wider UK, or much larger EU or global context, such reports, whether fact-based or more speculative, underline the importance of spectroscopy as a core photonic technology. The MATRIX report, in particular underscores photonics as an enabling technology, as realised by global partners, which can act as an intermediary technology for other areas of innovation.^215^

Focus can then be narrowed down from broader photonics to spectroscopy, including Raman devices. As with the portable spectrometer market ($1.7 billion USD, 2020),^39^ the wider worldwide sensing market is sizable and growing, currently standing above $2 billion USD (2024) with an 9% CAGR projected over the current decade,^39^ of which Raman devices will garner a significant share. At what scales these devices appear, however, remains to be seen. Often technological advancements do not find extensive use immediately – there is a lack of widespread need. This kind of ‘market lag’ phenomenon is not new. The prime example is Kodak, who developed the first digital camera in 1970s equipped with 100 × 100 pixel CCD and 20 s acquisition time. The 3 kg device however never took off – the memory and semiconductor (CMOS) technology was not ready for such a camera and the market had no need for it.^216^ While at one point we might have considered the development of miniaturised Raman systems too early – we were waiting for the market need to catch up, the requirement for remote sensing across the various application spheres means the time for small sensing devices is now.

As with other technologies, portable spectroscopic devices follow a familiar path of:

1. Development of the spectroscopic device and proposed applications,

2. Market exposure and feedback,

3. New applications, device refinement (calibrations, algorithms, databases *etc.*), and

4. Device optimisation/enhancement^3^

which includes not only portable mini-spectrometers, but those fully integrated to an on-chip level. This application/market-focused process is intricately linked to systems engineering approach, which as we suggest, many aspects of which can all be too easily overlooked by scientists working at early technology development stage.

When it comes to the smallest of Raman systems, their place may be very market-specific. For instance, in a food security context, using Raman spectroscopy for foodstuffs authenticity probably requires hand-held *i.e.* ∼1 kg devices with easy operation for non-spectroscopy skilled operatives. Contrariwise, applications in decentralised medicine or athletic performance monitoring may require wearable technology that needs to be as small as possible. Similarly, use of Raman sensors for monitoring of explosive precursors^217^ is likely to require significant miniaturisation for clandestine use.^218^ Any sensor that needs to be affixed in an elevated place or attached to a drone, say, also needs to be on the lighter side. At present there are multiple areas of research in need of miniaturisation, including Raman device integration into for example, robotic arms during surgeries, *in vivo* diagnostics, and substance identification at crime scenes.^219,220^ Adding spectrometers to consumer devices may add too much expense to the consumer without a suitable benefit in performance. One could imagine, for instance, a handheld pen spectrometer device that accurately indicates the freshness state of various foodstuffs, many of which are hard to pinpoint accurately (even with labels), however, this is a level of accuracy that is unlikely to interest most consumers at an elevated price point. Many application areas are still emerging. Within the UK, for instance, as we say, there is a push for more community-driven healthcare screening to relieve pressure from hospitals and studies on new biomarkers amid point-of-care testing continues to abound.^221^

In the short term, the point-of-care Raman market is unlikely to be driven by further incremental reductions in device size alone. Instead, the dominant trajectory appears to be towards task-specific, application-constrained systems, where optical performance is deliberately matched to a narrow diagnostic question rather than designed for broad analytical flexibility. In practice, this means accepting reduced spectral range or resolution where it does not materially impact diagnostic accuracy, in exchange for lower cost, faster acquisition and simpler regulatory validation. In healthcare settings in particular, PoC Raman devices are likely to succeed where they replace or de-risk an existing workflow, rather than where they attempt to introduce a new one. This favours devices targeted at well-defined use cases such as triage, screening or longitudinal monitoring, rather than general-purpose molecular analysis. As such, future PoC Raman systems may look less like miniaturised laboratory instruments and more like embedded diagnostic tools, co-designed with clinicians, regulators and end-users from the outset.

However, the bar for entering some sectors can be prohibitive for some investors. Healthcare devices, which are the focus for many Raman researchers, are higher risk and require a financial investment model that allows for a protracted product development.^28^ New medical devices now must conform to more stringent standards,^222^ which requires greater collaboration between developers and medical professionals. Within this application area specifically, a further problem is that the detection of biomarkers has been historically scattered and studies have lacked clinical validation. This may mean funders need to require a more concerted effort from researchers at the low TRL-range in order to facilitate effective device development at the later TRL stages.^221^

Elsewhere, the food supply has become increasingly globalised and this has mandated more portable sensing at various nodes in the chain to test the authenticity of what arrives on our supermarket shelves. Especially prominent events, such as the COVID19 pandemic (2019–2022) or the UK Horsemeat Scandal (2013)^223^ can be a real gamechangers for technology development, similar perhaps in how World War 2 resulted in significant strides in near-infrared devices. With climate change, environmental monitoring continues to be a concern, and the rise of AI and smart factories has also brought with it increased sensors needed for industrial quality control.

Moreover, different regions may have different strengths, and progress in the various areas that constitute making a Raman device could look different. The MATRIX report looking at photonics technologies points out that certain regions can have niches or competitive advantages due to existing capabilities or lack of competition in some photonics sectors. The report, for example, highlights the UK, South Korea and Australia as having particular expertise in spectroscopy and biophotonics (both $121 USD global market growth projection 2023–2030).^215^

### 9.8. Benchtop to backpocket

So, the nature of the device required, benchtop, handheld, or pocket-sized, depends on the application. Inevitably, there will be some trade-off in performance, cost, and size. Device size comparison studies, as have been performed in the context of near-infrared devices, would be useful.^224^ Portable Raman devices can align with Industry 4.0, also referred to as smart manufacturing in a manufacturing context, where sensing data can be collected continuously or at regular intervals from remote-controlled, automated internet-of-things (cloud-) connected sensors and analysed in real-time, assisting in industrial decision making, for *e.g.* quality control. Likely, devices of different forms and specifications will still be required, for example, increasing the numerical aperture of the pickup lens/fibres to increase the signal for especially low concentrations of weakly Raman-scattering analytes. We note, the smallest spectrometer devices currently being developed do not necessarily represent late technology readiness level products that are amenable to mass manufacture. As highlighted in ref. 42, the smallest footprint spectrometer (2022) on the tens μms-scale provides a resolution of 5–10 nm, insufficient for many applications. A large challenge will be to maintain spectral range and resolution, signal-to-noise, and adaptability to different kinds of sample forms.^3,77^ Similarly, on-chip Raman may not necessarily be the answer, for instance, larger drones can accommodate sensors in the 5 kg range. Wearable sensors will inevitably need to be small, but could be disposable SERS chips. Possibly, application of the smallest Raman sensors may be limited to very niche, albeit incredibly useful, applications, such as imaging within cells.^109^

**Fig. 9:**
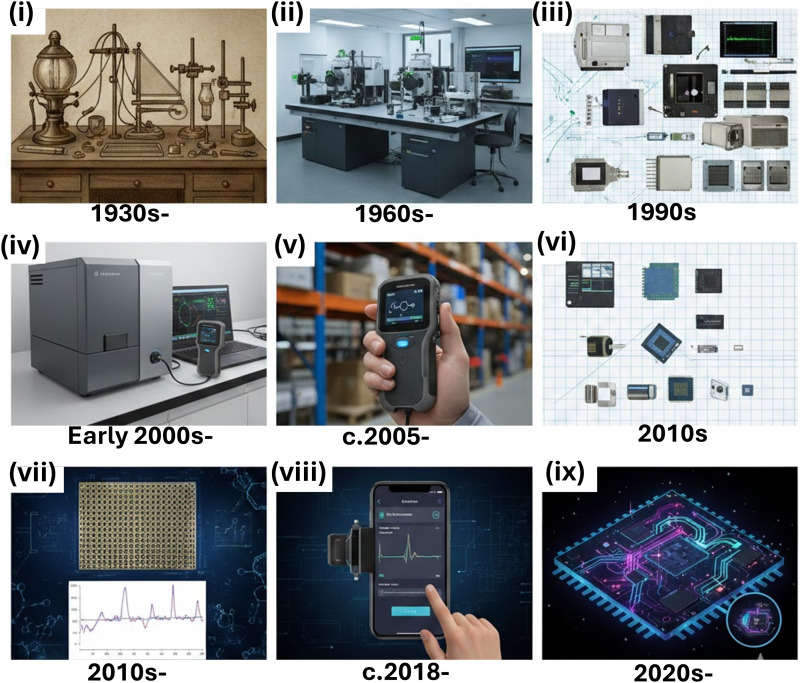
Past, present and future of Raman systems. A generative AI interpretation and pictorial representation of the various Raman spectroscopy systems. Top row (L–R): (i) Primitive Raman systems and (ii) large open-bench Raman systems with higher performance components, with (iii) progress in miniaturised constituent components. Middle row: (iv) Benchtop and portable Raman systems followed by (v) handheld Raman devices, (vi) facilitated by further downsizing of individual components. Bottom row: (vii) Progress in SERS substrates, especially those that are ‘rationally designed’ for quantitative analysis; (viii) research into smartphone-based detection, and (ix) focus on fully on-chip Raman devices alongside developments in photonic integrated circuits. Image generated with the assistance of Google Gemini.

Thus, predicting the precise trajectory for Raman miniaturisation is challenging (Fig. 9). It is hence important to keep talking to the analytical market to see what they want.^225^ Should the place for miniaturised Raman become clearer, there will be a need to keep adjusting to requirements for different applications: exact footprint and mass, resolution and wavelength range covered *etc.*, aligning with what an end user, perhaps even a consumer, would be willing to pay. It is now approaching 100 years since CV Raman discovered his eponymous effect (1928) and the progress that has been made has been remarkable where Raman spectroscopy has found a variety of applications, and spawned a range of derivative research areas, some of which, like SERS, have now become expansive fields of their own. Raman systems have seen impressive progress in miniaturisation. Perhaps the next century of Raman will deliver a range of devices, of different sizes and forms, to serve current application spaces and potentially a few new ones too.

## Author contributions

MH – sections 1–3, section 5 (On-chip Raman), section 6 (Incorporation of SERS-enhancing media), section 7.1 (Microfluidic devices), section 8.1 (Digital systems design), section 9 (Key challenges, market insights and future directions), Fig. 1, 2, 4, 6 and 8. TOC graphic. SI section material; conceptualisation; review and editing; post-review editing all sections. Project management and administration. PPK – section 4 (MEMS-based components in Raman miniaturisation); Fig. 5; TOC graphic. Post-review additions and editing: section 4; section 9.1. (MEMS, integration and fabrication challenges: maintaining performance). PPK also contributed to final manuscript proofing. EB – section 7 (Point-of-care Raman devices); Fig. 7. PGO – funding at UoB, manuscript review. Post-review additions to: section 8.1. (Digital systems design); section 9.4 (SERS outlook: analytical SERS and SERS-based devices); section 9.6. (The portable Raman device market). CPTMcP – section 8 (Systems engineering); Table 1, manuscript review and editing. RMB – funding at QUB; Management of Smart Nano NI Project.

## Conflicts of interest

The authors declare no conflict of interest. The authors have no formal relationships with any of the device manufacturers/companies named in the manuscript.

## Supplementary Material

LC-026-D5LC00836K-s001

## Data Availability

No primary research results, software or code have been included and no new data were generated or analysed as part of this review. Supplementary information (SI): the SI provides a comparison of charge-coupled device (CCD) and complementary metal oxide semiconductor (CMOS) sensors (Table S1) and a comparison of selected portable/carriable Raman systems (Table S2). See DOI: https://doi.org/10.1039/d5lc00836k.
