# Supplementary material for: Miniaturisation of Raman spectroscopy systems: from benchtop to backpocket
Source: Lab Chip. 2026 Mar 18;26(7):2112–45. doi: 10.1039/d5lc00836k (PMC12997533; doi:10.1039/d5lc00836k)
Supplement: LC-026-D5LC00836K-s001 [file LC-026-D5LC00836K-s001.pdf]

Supplementary Information (SI) for Lab on a Chip.
This journal is © The Royal Society of Chemistry 2026

– Supplementary Information –

Miniaturisation of Raman Spectroscopy Systems: From Benchtop to Backpocket

Mike Hardy[1,], Pooja P. Kanade[1], Emma Buchan[2,3], Pola Goldberg Oppenheimer[2,3], Cillian P.T. McPolin[1,4], and Robert M. Bowman[1].*

1. *Smart Nano NI, Centre for Quantum Materials and Technologies, School of Mathematics and Physics, Queen's University Belfast, Belfast BT7 1NN, UK.*
2. *Advanced Nano-Materials Structures and Applications Laboratories, School of Chemical Engineering, University of Birmingham, Birmingham B15 2TT, UK.*
3. *Healthcare Technologies Institute, Institute of Translational Medicine, Mindelsohn Way, Birmingham B15 2TH, UK*
4. *Digital Catapult Northern Ireland, 8 Lanyon Place, Belfast, BT1 3LP, UK.*

Feature	CCD Detectors	CMOS Detectors
**Sensitivity**	High, especially for low-light	High (now comparable to CCDs)
**Size**	Compact, but larger than CMOS	Smaller, highly integrated
**Noise**	Lower (cooled systems)	Improved with BSI and noise reduction
**Power Consumption**	Higher	Lower, more efficient
**Speed**	Moderate	High
**Cost**	Higher	Lower

Table S1. Comparison of Charge-Coupled Device (CCD) and Complementary Metal Oxide Semiconductor (CMOS) sensors. BSI=backside illumination [1]

Λ (nm)	Class	Spec. range	Spec. Res. FWHM	Mass (kg)	Notes 1	Notes 2
830 (450 mW)	3B	350-2000cm-1	<14cm-1 average	1.8	Medical apps of SORS 'interesting'	Defocussed to probe maximal area / offset detector for SORS. 257mm depth.
785 & 852 'DuoLaser' two laser system.	1M	300-3200cm-1	Typically <1cm-1 (300-1900cm-1)	'Handheld'		SSE for flu rejection. This uses variable temperature to remove background.
785 / 1064	enclosed	? 3000cm-1 +	1.5-4cm-1	benchtop	confocal	Can incorporate FT-Raman @ 1064nm if desired
785nm	Glasses necessary (provided)	200-2750cm-1	4cm-1	2.5kg This is a small Raman spectrometer with fibre optic attachment that inc. optics.	Variable performance comes down to different optics and cooling options.	
532nm version	50mw-70mW	200-4500cm-1	9cm-1 and under			
785 (350mW)		250-2875cm-1	8-10.5cm-1	0.9kg Handheld	NA=0.33	Solo software. Measurement Accessories Vial holder, universal tablet holder, cuvette holder<br>Qualitative/semi-quant
785 (450mW)	Probes 3B laser	100- 3250cm-1	6.5cm-1 mean	benchtop H: 7.7 cm W: 25 cm D: 25 cm		Fiber BallProbe® probe<br>Raman suite softeare<br>Quantiative Raman
785 (<100mW)	3B	400-2300cm-1	0.6nm 8-10cm-1	0.7kg	Library of illicit materials.	
785 (340mW)	fibre-coupled	150-2800cm-1	<6cm-1@912nm	9kg 'Portable Raman')	-25oC cooled CCD	Video uscope w/ xyz stage
785 (340mW)	fibre-coupled	65-2800cm-1	3.5cm-1@912nm	5kg ('Portable Raman')	-2oC cooled CCD	Algorithm for flu rej. 'Algorithm might not work so well at 532nm w/ lot of flu.' Orbital raster scanning for averaging. 1064nm 532nm lasers also available
785 (300mW)		176- 2900cm-1		1kg		Libraries: Narcotics, Explosives*, Pharmaceutical Drugs, Cutting Agents, Precursors, Toxic & Common Chemicals, and More 1064nm versions also available.

Table S2. Comparison of selected portable/carriable Raman systems. L-R: Vendor, Model name, Laser Wavelength, Laser class (or safety information otherwise), Spectral range (wavenumbers), Spectral Resolution (wavenumbers or nanometres), Mass, Note/Other details. FWHM=full width half maximum. Each colour represents a different Raman vendor.

References

[1] OpenAI ChatGPT: Scholar AI GPT [Large language model]. https://chat.openai.com
